# Nanopore sequencing and assembly of a human genome with ultra-long reads

**DOI:** 10.1038/nbt.4060

**Published:** 2018-01-29

**Authors:** Miten Jain, Sergey Koren, Karen H Miga, Josh Quick, Arthur C Rand, Thomas A Sasani, John R Tyson, Andrew D Beggs, Alexander T Dilthey, Ian T Fiddes, Sunir Malla, Hannah Marriott, Tom Nieto, Justin O'Grady, Hugh E Olsen, Brent S Pedersen, Arang Rhie, Hollian Richardson, Aaron R Quinlan, Terrance P Snutch, Louise Tee, Benedict Paten, Adam M Phillippy, Jared T Simpson, Nicholas J Loman, Matthew Loose

**Affiliations:** 1 UC Santa Cruz Genomics Institute, University of California, Santa Cruz, California USA; 2 Genome Informatics Section, Computational and Statistical Genomics Branch, National Human Genome Research Institute, Bethesda, Maryland USA; 3 Institute of Microbiology and Infection, University of Birmingham, Birmingham, UK; 4 Department of Human Genetics, University of Utah, Salt Lake City, Utah USA; 5 USTAR Center for Genetic Discovery, University of Utah, Salt Lake City, Utah USA; 6 Michael Smith Laboratories and Djavad Mowafaghian Centre for Brain Health, University of British Columbia, Vancouver, Canada; 7 Surgical Research Laboratory, Institute of Cancer & Genomic Science, University of Birmingham, UK; 8 DeepSeq, School of Life Sciences, University of Nottingham, UK; 9 Norwich Medical School, University of East Anglia, Norwich, UK; 10 Department of Biomedical Informatics, University of Utah, Salt Lake City, Utah USA; 11 Ontario Institute for Cancer Research, Toronto, Canada; 12 Department of Computer Science, University of Toronto, Toronto, Canada

**Keywords:** Genomics, Next-generation sequencing, DNA sequencing

## Abstract

We report the sequencing and assembly of a reference genome for the human GM12878 Utah/Ceph cell line using the MinION (Oxford Nanopore Technologies) nanopore sequencer. 91.2 Gb of sequence data, representing ∼30× theoretical coverage, were produced. Reference-based alignment enabled detection of large structural variants and epigenetic modifications. *De novo* assembly of nanopore reads alone yielded a contiguous assembly (NG50 ∼3 Mb). We developed a protocol to generate ultra-long reads (N50 > 100 kb, read lengths up to 882 kb). Incorporating an additional 5× coverage of these ultra-long reads more than doubled the assembly contiguity (NG50 ∼6.4 Mb). The final assembled genome was 2,867 million bases in size, covering 85.8% of the reference. Assembly accuracy, after incorporating complementary short-read sequencing data, exceeded 99.8%. Ultra-long reads enabled assembly and phasing of the 4-Mb major histocompatibility complex (MHC) locus in its entirety, measurement of telomere repeat length, and closure of gaps in the reference human genome assembly GRCh38.

**Supplementary information:**

The online version of this article (doi:10.1038/nbt.4060) contains supplementary material, which is available to authorized users.

## Abstract (web summary)

A human genome is sequenced and assembled *de novo* using a pocket-sized nanopore device.

**Supplementary information:**

The online version of this article (doi:10.1038/nbt.4060) contains supplementary material, which is available to authorized users.

## Main

The human genome is used as a yardstick to assess performance of DNA sequencing instruments^1,2,3,4,5^. Despite improvements in sequencing technology, assembling human genomes with high accuracy and completeness remains challenging. This is due to size (∼3.1 Gb), heterozygosity, regions of GC% bias, diverse repeat families, and segmental duplications (up to 1.7 Mbp in size) that make up at least 50% of the genome^6^. Even more challenging are the pericentromeric, centromeric, and acrocentric short arms of chromosomes, which contain satellite DNA and tandem repeats of 3–10 Mb in length^7,8^. Repetitive structures pose challenges for *de novo* assembly using “short read” sequencing technologies, such as Illumina's. Such data, while enabling highly accurate genotyping in non-repetitive regions, do not provide contiguous *de novo* assemblies. This limits the ability to reconstruct repetitive sequences, detect complex structural variation, and fully characterize the human genome.

Single-molecule sequencers, such as Pacific Biosciences' (PacBio), can produce read lengths of 10 kb or more, which makes *de novo* human genome assembly more tractable^9^. However, single-molecule sequencing reads have significantly higher error rates compared with Illumina sequencing. This has necessitated development of *de novo* assembly algorithms and the use of long noisy data in conjunction with accurate short reads to produce high-quality reference genomes^10^. In May 2014, the MinION nanopore sequencer was made available to early-access users^11^. Initially, the MinION nanopore sequencer was used to sequence and assemble microbial genomes or PCR products^12,13,14^ because the output was limited to 500 Mb to 2 Gb of sequenced bases. More recently, assemblies of eukaryotic genomes including yeasts, fungi, and *Caenorhabditis elegans* have been reported^15,16,17^.

Recent improvements to the protein pore (a laboratory-evolved *Escherichia coli* CsgG mutant named R9.4), library preparation techniques (1D ligation and 1D rapid), sequencing speed (450 bases/s), and control software have increased throughput, so we hypothesized that whole-genome sequencing (WGS) of a human genome might be feasible using only a MinION nanopore sequencer^17,18,19^.

We report sequencing and assembly of a reference human genome for GM12878 from the Utah/CEPH pedigree, using MinION R9.4 1D chemistry, including ultra-long reads up to 882 kb in length. GM12878 has been sequenced on a wide variety of platforms, and has well-validated variation call sets, which enabled us to benchmark our results^20^.

## Results

### Sequencing data set

**Figure 1:**
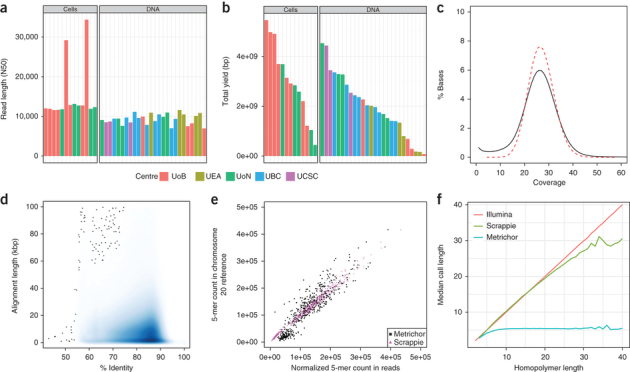
Summary of data set. (**a**) Read length N50s by flow cell, colored by sequencing center. Cells: DNA extracted directly from cell culture. DNA: pre-extracted DNA purchased from Coriell. UoB, Univ. Birmingham; UEA, Univ. East Anglia; UoN, Univ. Nottingham; UBC, Univ. British Columbia; UCSC, Univ. California, Santa Cruz. (**b**) Total yield per flow cell grouped as in **a**. (**c**) Coverage (black line) of GRCh38 reference compared to a Poisson distribution. The depth of coverage of each reference position was tabulated using samtools depth and compared with a Poisson distribution with lambda = 27.4 (dashed red line). (**d**) Alignment identity compared to alignment length. No length bias was observed, with long alignments having the same identity as short ones. (**e**) Correlation between 5-mer counts in reads compared to expected counts in the chromosome 20 reference. (**f**) Chromosome 20 homopolymer length versus median homopolymer base-call length measured from individual Illumina and nanopore reads (Scrappie and Metrichor). Metrichor fails to produce homopolymer runs longer than ∼5 bp. Scrappie shows better correlation for longer homopolymer runs, but tends to overcall short homopolymers (between 5 and 15 bp) and undercall long homopolymers (>15 bp). Plot noise for longer homopolymers is due to fewer samples available at that length.

Five laboratories collaborated to sequence DNA from the GM12878 human cell line. DNA was sequenced directly (avoiding PCR), thus preserving epigenetic modifications such as DNA methylation. 39 MinION flow cells generated 14,183,584 base-called reads containing 91,240,120,433 bases with a read N50 (the read length such that reads of this length or greater sum to at least half the total bases) of 10,589 bp (Supplementary Tables 1–4). Ultra-long reads were produced using 14 additional flow cells. Read lengths were longer when the input DNA was freshly extracted from cells compared with using Coriell-supplied DNA (Fig. 1a). Average yield per flow cell (2.3 Gb) was unrelated to DNA preparation methods (Fig. 1b). 94.15% of reads had at least one alignment to the human reference (GRCh38) and 74.49% had a single alignment over 90% of their length. Median coverage depth was 26-fold, and 96.95% (3.01/3.10 Gbp) of bases of the reference were covered by at least one read (Fig. 1c). The median identity of reads was 84.06% (82.73% mean, 5.37% s.d.). No length bias was observed in the error rate with the MinION (Fig. 1d).

### Base-caller evaluation

The base-calling algorithm used to decode raw ionic current signal can affect sequence calls. To analyze this effect we used reads mapping to chromosome 20 and compared base-calling with Metrichor (an LSTM-RNN base-caller) and Scrappie, an open-source transducer neural network (Online Methods). Of note, we observed that a fraction of the Scrappie output (4.7% reads, 14% bases) was composed of low-complexity sequence (Supplementary Fig. 1), which we removed before downstream analysis.

To assess read accuracy we realigned reads from each base-caller using a trained alignment model^21^. Alignments generated by the Burrows–Wheeler Aligner Maximal Exact Matches (BWA-MEM) were chained such that each read had at most one maximal alignment to the reference sequence (scored by length). The chained alignments were used to derive the maximum likelihood estimate of alignment model parameters^22^, and the trained model used to realign the reads. The median identity after realignment for Metrichor was 82.43% and for Scrappie, 86.05%. We observed a purine-to-purine substitution bias in chained alignments where the model was not used (Supplementary Fig. 2). The alignments produced by the trained model showed an improved substitution error rate, decreasing the overall transversion rate, but transition errors remained dominant.

To measure potential bias at the *k*-mer level, we compared counts of 5-mers in reads derived from chromosome 20. In Metrichor reads, the most underrepresented 5-mers were A/T-rich homopolymers. The most overrepresented *k*-mers were G/C-rich and non-homopolymeric (Supplementary Table 5). By contrast, Scrappie showed no underrepresentation of homopolymeric 5-mers and had a slight overrepresentation of A/T homopolymers. Overall, Scrappie showed the lowest *k*-mer representation bias (Fig. 1e). The improved homopolymer resolution of Scrappie was confirmed by inspection of chromosome 20 homopolymer calls versus the human reference (Fig. 1f and Supplementary Fig. 3)^23^. Despite this reduced bias, whole-genome assembly and analyses proceeded with Metrichor reads, since Scrappie was still in early development at the time of writing.

### *De novo* assembly of nanopore reads

We carried out a *de novo* assembly of the 30× data set with Canu^24^ (Table 1). This assembly comprised 2,886 contigs with an NG50 contig size of 3 Mbp (NG50, the longest contig such that contigs of this length or greater sum to at least half the haploid genome size). The identity to GRCh38 was estimated as 95.20%. Canu was fourfold slower on the Nanopore data compared to a random subset of equivalent coverage of PacBio data requiring ∼62K CPU hours. The time taken by Canu increased when the input was nanopore sequence reads because of systematic error in the raw sequencing data leading to reduced accuracy of the Canu-corrected reads, an intermediate output of the assembler. Corrected PacBio reads are typically >99% identical to the reference; our reads averaged 92% identity to the reference after correction (Supplementary Fig. 1b).

**Table 1:** Summary of assembly statistics

Assembly	Polishing	Contigs	No. bases (Mbp)	Max contig (kb)	NG50 (kb)	GRCh38 identity (%)	GM12878 identity (%)
WGS Metrichor	N/A	2,886	2,646.01	27,160	2,964	95.20	95.74
	Pilon x2		2,763.18	28,413	3,206	99.29	99.88
Chr 20 Metrichor	N/A	85	57.83	7,393	3,047	94.90	95.50
	Nanopolish		60.35	7,667	5,394	98.84	99.24
	Pilon x2		60.58	7,680	5,423	99.33	99.89
	Nano + Pilon x2		60.76	7,698	5,435	99.64	99.95
Chr 20 Scrappie	N/A	74	59.39	8,415	2,643	97.43	97.80
	Nanopolish		60.15	8,521	2,681	99.12	99.44
	Pilon x2		60.36	8,541	2,691	99.64	99.95
	Nano + Pilon x2		60.34	8,545	2,691	99.70	99.96

Summary of assembly statistics. Whole genome assembly (WGA) was performed with reads base-called by Metrichor. Chromosome 20 was assembled with reads produced byMetrichor and Scrappie. All data sets contained 30× coverage of the genome/chromosome. The GRCh38 identities were computed based on 1-1 alignments to the GRCh38 reference including alt sites. A GM12878 reference was estimated using an independent sequencing data set^20^.

We aligned assembled contigs to the GRCh38 reference and found that our assembly was in agreement with previous GM12878 assemblies (Supplementary Fig. 4)^25^. The number of structural differences (899) that we identified between GM12878 and GRCh38 was similar to that of a previously published PacBio assembly of GM12878 (692) and of other human genome assemblies^5,24^, but with a higher than expected number of deletions, due to consistent truncation of homopolymer and low-complexity regions (Supplementary Fig. 5 and Supplementary Table 6). Consensus identity of our assembly with GRCh38 was estimated to be 95.20% (Table 1). However, GRCh38 is a composite of multiple human haplotypes, so this is a lower bound on accuracy. Comparisons with independent Illumina data from GM12878 yielded a higher accuracy estimate of 95.74%.

Despite the low consensus accuracy, contiguity was good. For example, the assembly included a single ∼3-Mbp contig that had all class I human leukocyte antigens (HLA) genes from the major histocompatibility complex (MHC) region on chromosome 6, a region notoriously difficult to assemble using short reads. The more repetitive class II HLA gene locus was fragmented but most genes were present in a single contig.

### Genome polishing

To improve the accuracy of our assembly we mapped previously generated whole-genome Illumina data (SRA: ERP001229) to each contig using BWA-MEM and corrected errors using Pilon. This improved the estimated accuracy of our assembly to 99.29% versus GRCh8 and 99.88% versus independent GM12878 sequencing (Table 1 and Supplementary Fig. 6)^26^. This estimate is a lower bound as true heterozygous variants and erroneously mapped sequences decrease identity. Recent PacBio assemblies of mammalian genomes that were assembled *de novo* and polished with Illumina data exceed 99.95%^9,27^. Pilon cannot polish regions that have ambiguous short-read mappings, that is, in repeats. We also compared the accuracy of our polished assembly in regions with expected coverage versus those that had low-quality mappings (either lower coverage or higher than expected coverage with low mapping quality) versus GRCh38. When compared to GRCh38, accuracy in well-covered regions increased to 99.32% from the overall accuracy of 99.29%, while the poorly covered regions accuracy dropped to 98.65%.

For further evaluation of our assembly, we carried out comparative annotation before and after polishing (Supplementary Table 7). 58,338 genes (19,436 coding, 96.4% of genes in GENCODE V24, 98.2% of coding genes) were identified representing 179,038 transcripts in the polished assembly. Reflecting the assembly's high contiguity, only 857 (0.1%) of genes were found on two or more contigs.

Alternative approaches to improve assembly accuracy using different base-callers and exploiting the ionic current signal were attempted on a subset of reads from chromosome 20. Assembly consensus improvement using raw output is commonly used when assembling single-molecule data. To quantify the effect of base-calling on the assembly, we reassembled the read sets from Metrichor and Scrappie with the same Canu parameters used for the whole-genome data set. While all assemblies had similar contiguity, using Scrappie reads improved accuracy from 95.74% to 97.80%. Signal-level polishing of Scrappie-assembled reads using nanopolish increased accuracy to 99.44%, and polishing with Illumina data brought the accuracy up to 99.96% (Table 1).

### Analysis of sequences not in the assembly

To investigate sequences omitted from the primary genome analysis, we assessed 1,425 contigs filtered from Canu due to low coverage, or contigs that were single reads with many shorter reads within (26 Mbp), or corrected reads not incorporated into contigs (10.4 Gbp). Most sequences represented repeat classes, for example, long interspersed nuclear elements (LINEs) and short interspersed nuclear elements (SINEs) (Supplementary Fig. 7), observed in similar proportion in the primary assembly, with the exception of satellite DNAs known to be enriched in human centromeric regions. These satellites were enriched 2.93× in the unassembled data and 7.9× in the Canu-filtered contigs. We identified 56 assembled contigs containing centromere repeat sequences specific to each of the 22 autosomes and X chromosome. The largest assembled satellite in these contigs is a 94-kbp tandem repeat specific to centromere 15 (D15Z1, tig00007244).

### SNP and SV genotyping

**Figure 2:**
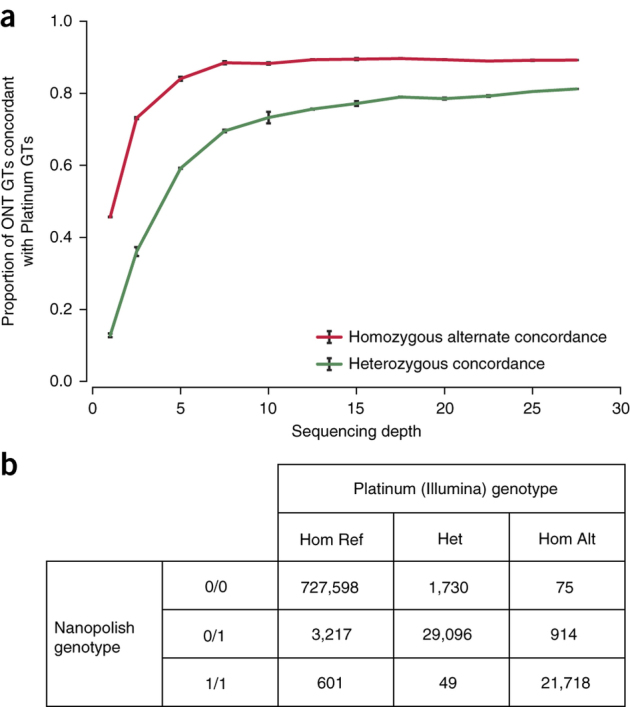
Structural variation and SNP genotyping. (**a**) Structural variant genotyping sensitivity using Oxford Nanopore Technologies (ONT) reads. Genotypes (GTs) were inferred for a set of 2,414 SVs using both Oxford Nanopore and Platinum Genomes (Illumina) alignments. Using alignments randomly subsampled to a given sequencing depth (*n* = 3), sensitivity was calculated as the proportion of ONT-derived genotypes that were concordant with Illumina-derived genotypes. (**b**) Confusion matrix for genotype-calling evaluation. Each cell contains the number of 1000 Genome sites for a particular nanopolish/platinum genotype combination.

Using SVTyper^28^ and Platinum Illumina WGS alignments, we genotyped 2,414 GM12878 structural variants (SVs), which were previously identified using LUMPY and validated with PacBio and/or Moleculo reads^29^. We then genotyped the same SVs using alignments of our nanopore reads from the 30×-coverage data set and a modified version of SVTyper. We measured the concordance of genotypes at each site in the Illumina- and nanopore-derived data, deducing the sensitivity of SV genotyping as a function of nanopore sequencing depth (Fig. 2a). When all 39 flow cells were used, nanopore data recovered 91% of high-confidence SVs with a false-positive rate of 6%. Illumina and nanopore genotypes agreed at 81% of heterozygous sites and 90% of homozygous alternate sites. Genotyping heterozygous SVs using nanopore alignments was limited when homopolymer stretches occur at the breakpoints of these variants (Supplementary Fig. 8a). We determined Illumina, nanopore, and PacBio genotype concordance at a set of 2,192 deletions common to our high-confidence set and a genotyped SV call set derived from PacBio sequencing of GM12878 (refs. 5, 30). PacBio and Illumina genotypes agreed at 94% of heterozygous and 79% of homozygous alternate deletions; nanopore and Illumina genotypes agreed at 90% of heterozygous and 90% of homozygous alternate sites; nanopore and PacBio genotypes agreed at 91% of heterozygous and 76% of homozygous alternate sites. Nearly a quarter (44) of the homozygous alternate sites at which PacBio and Illumina genotypes disagreed overlapped SINEs or LINEs. By manual inspection in the integrative genomics viewer (IGV)^31^, we observed that sequencing reads were spuriously aligned at these loci and likely drove the discrepancy in predicted genotypes (Supplementary Fig. 8b).

We evaluated nanopore data for calling genotypes at known single-nucleotide polymorphisms (SNPs) using the ionic current by calling genotypes at non-singleton SNPs on chromosome 20 from phase 3 of the 1000 Genomes^32^ and comparing these calls to Illumina Platinum Genome calls (Fig. 2b). 99.16% of genotype calls were correct (778,412 out of 784,998 sites). This result is dominated by the large number of homozygous reference sites. If we assess accuracy by the fraction of correctly called variant sites (heterozygous or homozygous non-reference), the accuracy of our caller is 91.40% (50,814 out of 55,595), with the predominant error being miscalling sites labeled homozygous in the reference as heterozygous (3,217 errors). Genotype accuracy, when only considering sites annotated as variants in the platinum call set, is 94.83% (50,814 correct out of 53,582).

### Detection of epigenetic 5-methylcytosine modification

**Figure 3:**
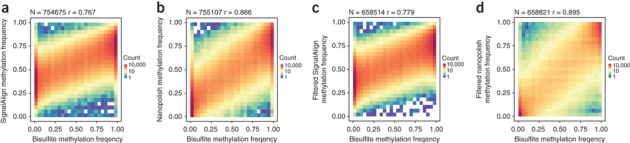
Methylation detection using signal-based methods. (**a**) SignalAlign methylation probabilities compared to bisulfite sequencing frequencies at all called sites. (**b**) Nanopolish methylation frequencies compared to bisulfite sequencing at all called sites. (**c**) SignalAlign methylation probabilities compared to bisulfite sequencing frequencies at sites covered by at least ten reads in the nanopore and bisulfite data sets; reads were not filtered for quality. (**d**) Nanopolish methylation frequencies compared to bisulfite sequencing at sites covered by at least ten reads in the nanopore and bisulfite data sets. A minimum log-likelihood threshold of 2.5 was applied to remove ambiguous reads. *N* = sample size, *r* = Pearson correlation coefficient.

Changes in the ionic current when modified and unmodified bases pass through the MinION nanopores enable detection of epigenetic marks^33,34^. We used nanopolish and SignalAlign to map 5-methylcytosine at CpG dinucleotides as detected in our sequencing reads against chromosome 20 of the GRCh38 reference^35,36^. Nanopolish outputs a frequency of reads calling a methylated cytosine, and SignalAlign outputs a marginal probability of methylation summed over reads. We compared the output of both methods to published bisulfite sequencing data from the same DNA region (ENCFF835NTC). Good concordance of our data with the published bisulfite sequencing was observed; the *r*-values for nanopolish and SignalAlign were 0.895 and 0.779, respectively (Fig. 3 and Supplementary Figs. 9 and 10).

### Ultra-long reads improve phasing and assembly contiguity

**Figure 4:**
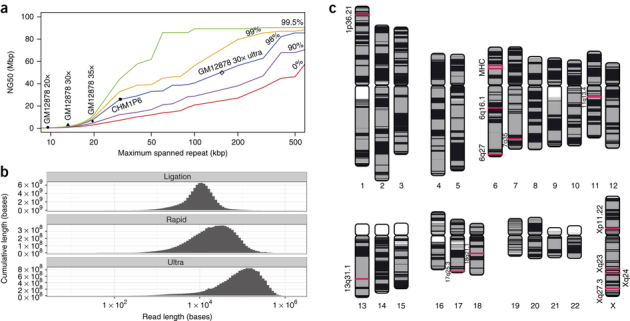
Repeat modeling and assembly. (**a**) A model of expected NG50 contig size when correctly resolving human repeats of a given length and identity. The *y* axis shows the expected NG50 contig size when repeats of a certain length (*x* axis) or sequence identity (colored lines) can be consistently resolved. Nanopore assembly contiguity (GM12878 20×, 30×, 35×) is currently limited by low coverage of long reads and a high error rate, making repeat resolution difficult. These assemblies approximately follow the predicted assembly contiguity. The *projected* assembly contiguity using 30 × of ultra-long reads (GM12878 30× ultra) exceeds 30 Mbp. A recent assembly of 65 × PacBio P6 data with an NG50 of 26 Mbp is shown for comparison (CHM1 P6). (**b**) Yield by read length (log_10_) for ligation, rapid and ultra-long rapid library preparations. (**c**) Chromosomes plot illustrating the contiguity of the nanopore assembly boosted with ultra-long reads. Contig and alignment boundaries, not cytogenetic bands, are represented by a color switch, so regions of continuous color indicate regions of contiguous sequence. White areas indicate unmapped sequence, usually caused by N's in the reference genome. Regions of interest, including the 12 50+ kb gaps in GRCh38 closed by our assembly as well as the MHC (16 Mbp), are outlined in red.

We modeled the contribution of read length to assembly quality, predicting that ultra-long read data sets (N50 >100 kb) would substantially improve assembly contiguity (Fig. 4a). We developed a method to produce ultra-long reads by saturating the Oxford Nanopore Rapid Kit with high molecular weight DNA. In so doing we generated an additional 5× coverage (Supplementary Fig. 11). Two additional standard protocol flow cells generated a further 2× coverage and were used as controls for software and base-caller versions. The N50 read length of the ultra-long data set was 99.7 kb (Fig. 4b). Reads were impossible to align efficiently at first, because aligner algorithms are optimized for short reads. Further, CIGAR strings generated by ultra-long reads do not fit in the BAM format specification, necessitating the use of SAM or CRAM formats only (https://github.com/samtools/hts-specs/issues/40). Instead, we used GraphMap^37^ to align ultra-long reads to GRCh38, which took >25K CPU hours (Supplementary Table 8). Software optimized for long reads, including NGM-LR^38^ and Minimap2 (ref. 39), were faster: Minimap2 took 60 CPU hours. More than 80% of bases were in sequences aligned over 90% of their length with GraphMap and more than 60% with minimap2. Median alignment identity was 81% (83 with minimap2), slightly lower than observed for the control flow cells (83.46%/84.64%) and the original data set (83.11%/84.32%). The longest full-length mapped read in the data set (aligned with GraphMap) was 882 kb, corresponding to a reference span of 993 kb.

The addition of 5× coverage ultra-long reads more than doubled the previous assembly NG50 to 6.4 Mbp and resolved the MHC locus into a single contig (Fig. 4c). In comparison, a 50× PacBio GM12878 data set with average read length of 4.5 kb assembled with an NG50 contig size of 0.9 Mbp^5^. Newer PacBio assemblies of a human haploid cell line, with mean read lengths greater than 10 kb, have reached contig NG50s exceeding 20 Mbp at 60× coverage^25^. We subsampled this data set to a depth equivalent to ours (35×) and assembled, resulting in an NG50 of 5.7 Mbp, with the MHC split into >2 contigs. The PacBio assembly is less contiguous, despite a higher average read length and simplified haploid genome.

**Figure 5:**
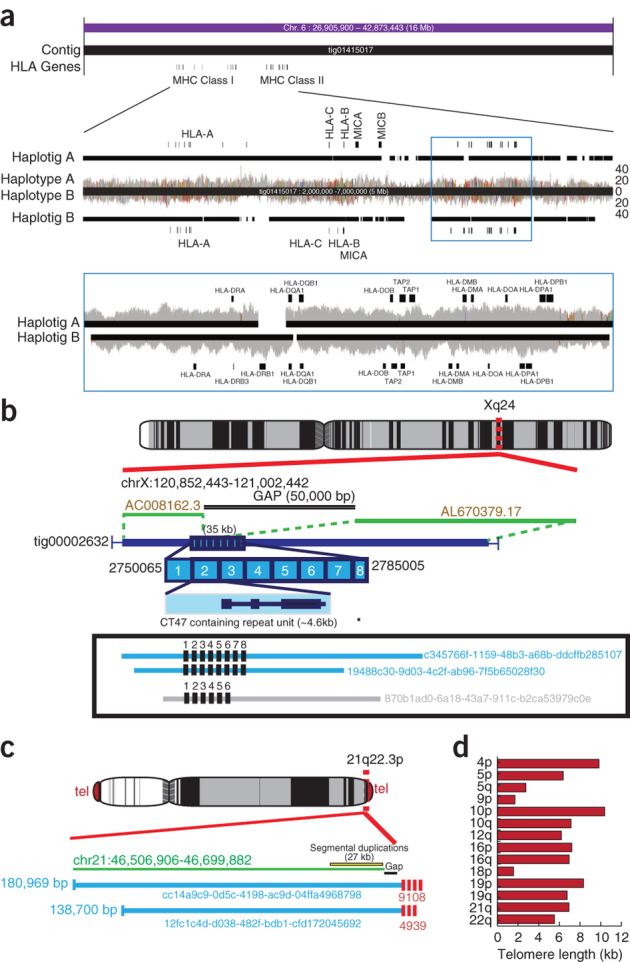
Ultra-long reads, assembly, and telomeres. (**a**) A 16-Mbp ultra-long read contig and associated haplotigs are shown spanning the full MHC region. MHC Class I and II regions are annotated along with various HLA genes. Below this contig, the MHC region is enlarged, showing haplotype A and B coverage tracks for the phased nanopore reads. Nanopore reads were aligned back to the polished Canu contig, with colored lines indicating a high fraction of single-nucleotide discrepancies in the read pileups (as displayed by the IGV^31^ browser). The many disagreements indicate the contig is a mosaic of both haplotypes. The haplotig A and B tracks show the result of assembling each haplotype read set independently. Below this, the MHC class II region is enlarged, with haplotype A and B raw reads aligned to their corresponding, unpolished haplotigs. The few consensus disagreements between raw reads and haplotigs indicate successful partitioning of the reads into haplotypes. (**b**) An unresolved, 50-kb bridged scaffold gap on Xq24 remains in the GRCh38 assembly (adjacent to scaffolds AC008162.3 and AL670379.17, shown in green). This gap spans a ∼4.6-kb tandem repeat containing cancer/testis gene family 47 (CT47). This gap is closed by assembly (contig: tig00002632) and has eight tandem copies of the repeat, validated by alignment of 100 kb+ ultra-long reads also containing eight copies of the repeat (light blue with read name identifiers). One read has only six repeats, suggesting the tandem repeated units are variable between homologous chromosomes. (**c**) Ultra-long reads can predict telomere length. Two 100 kb+ reads that map to the subtelomeric region of the chromosome 21 q-arm, each containing 4.9–9.1 kb of the telomeric (TTAGGG_ repeat). (**d**) Telomere length estimates showing variable lengths between non-homologous chromosomes.

In addition to assembling the MHC into a single contig, the ultra-long MinION reads enabled the contiguous MHC to be haplotype phased. Due to the limited depth of nanopore reads, heterozygous SNPs were called using Illumina data and then phased using the ultra-long nanopore reads to generate two pseudo-haplotypes, from which MHC typing was performed using the approach of Dilthey *et al*.^40^ (Fig. 5a). Some gaps were introduced during haplotig (contigs with the same haplotype) assembly, owing to low phased-read coverage—for example, *HLA-DRB3* was left unassembled on haplotype A—but apart from one *HLA-DRB1* allele, sample HLA types wererecovered almost perfectly with an edit distance of 0–1 for true allele versus called allele (Supplementary Table 9). Analysis of parental (GM12891, GM12892) HLA types confirmed the absence of switch errors between the classical HLA typing genes. To our knowledge, this is the first time the MHC has been assembled and phased over its full length in a diploid human genome.

Already published single-molecule human genome assemblies contain multiple contigs that span the MHC^5,41,42^ and phasing has not been attempted. Instead, MHC surveys have focused on homozygous cell lines^43^.

### Ultra-long reads close gaps in the human reference genome

Large (>50 kb) bridged scaffold gaps remain unresolved in the reference human genome assembly (GRCh38). These breaks in the assembly span tandem repeats and/or long tracts of segmental duplications^44^. Using sequence from our *de novo*–assembled contigs, we were able to close 12 gaps, each of which was more than 50 kb in the reference genome. We then looked for individual ultra-long reads that spanned gaps, and matched the sequence closure for each region as predicted by the assembly (Supplementary Table 10).

The gap closures enabled us to identify 83,980 bp of previously unknown euchromatic sequence. For example, an unresolved 50-kbp scaffold gap on Xq24 marks the site of a human-specific tandem repeat that contains a cancer/testis gene family, known as CT47 (refs. 45, 46). This entire region is spanned by a single contig in our final assembly (tig00002632). Inspection of this contig using hidden Markov model (HMM) profile modeling of an individual repeat unit containing the *CT47A11* gene (GRCh38 chrX:120932333–120938697) suggests that there is an array of eight tandem copies of the CT47 repeat (Fig. 5b). In support of this finding, we identified three ultra-long reads that together traversed the entire tandem array (Fig. 5b); two reads provide evidence for an array of eight repeat copies and one read supports six copies, suggesting heterozygosity.

### Telomere repeat lengths

FISH (fluorescent *in situ* hybridization) estimates and direct cloning of telomeric DNA suggests that telomere repeats (TTAGGG) extend for multiple kilobases at the ends of each chromosome^47,48^. Using HMM profile modeling of the published telomere tract of repeats (M19947.1), we identified 140 ultra-long reads that contained the TTAGGG tandem repeat (Supplementary Table 11). Sequences next to human telomeres are enriched in intra- and interchromosomal segmental duplications, which makes it difficult to map ultra-long reads directly to the chromosome assemblies. However, we were able to map 17/140 ultra-long reads to specific chromosome subtelomeric regions. We analyzed the mapped regions by identifying the junction or the start of the telomeric array on 17 ultra-long reads, and annotating all TTAGGG-repeat sequences to the end of the read to estimate telomeric repeat length. For example, two reads that only mapped to chromosome 21q indicate that there are 9,108 bp of telomeric repeats. Overall, we found evidence for telomeric arrays that span 2–11 kb within 14 subtelomeric regions for GM12878 (Fig. 5c,d and Supplementary Table 11).

## Discussion

We report sequencing and assembly of a human genome with 99.88% accuracy and an NG50 of 6.4 Mb using unamplified DNA and nanopore reads followed by short-read consensus improvement. At 30× coverage we have produced the most contiguous assembly of a human genome to date, using only a single sequencing technology and the Canu assembler^23^. Consistent with the view that the underlying ionic raw current contains additional information, signal-based polishing^14^ improved the assembly accuracy to 99.44%. Finally, we report that combining signal-based polishing and short-read (Illumina) correction^26^ gave an assembly accuracy of 99.96%, which is similar to metrics for other mammalian genomes^9^.

Here we report that read lengths produced by the MinION nanopore sequencer were dependent on the input fragment length. We found that careful preparation of DNA in solution using classical extraction and purification methods can yield extremely long reads. The longest read lengths were achieved using the transposase-based rapid library kit in conjunction with methods of DNA extraction designed to mitigate shearing. We produced 5× coverage with ultra-long reads, and used this data set to augment our initial assembly. The final 35× coverage assembly has an NG50 of 6.4 Mb. Based on modeling we predict that 30× of ultra-long reads alone would result in an assembly with a contig NG50 in excess of 40 Mb, approaching the contiguity of the current human reference (Fig. 4c). We posit that there may be no intrinsic read-length limit for pore-based sequencers, other than from physical forces that lead to DNA fragmentation in solution. Therefore, there is scope to further improve the read-length results obtained here, perhaps through solid-phase DNA extraction and library preparation techniques, such as agar encasement.

The increased single-molecule read length that we report here, obtained using a MinION nanopore sequencer, enabled us to analyze regions of the human genome that were previously intractable with state-of-the-art sequencing methods. For example, we were able to phase megabase regions of the human genome in single contigs, to more accurately estimate telomere lengths, and to resolve complex repeat regions. Phasing of 4- to 5-Mb scaffolds through the MHC has recently been reported using a combination of sequencing and genealogical data^49^. However, the resulting assemblies contained multiple gaps of unknown sequences. We phased the entire MHC, and reconstructed both alleles. Development of tools to automate phasing from nanopore assemblies is now needed.

We also wrote custom software/algorithms (poredb) to track the large number of reads, store each read as an individual file, and enable use of cloud-based pipelines for our analyses.

Our proof-of-concept demonstration of human genome sequencing using a MinION nanopore sequencer reveals the potential of this approach, but identifies specific challenges for future projects. Improvements in real-time base-calling are needed to simplify the workflow. More compact and convenient formats for storing raw and base-called data are urgently required, ideally employing a standardized, streaming compatible serialization format such as BAM/CRAM.

With ultra-long reads we found the longest reads exceeded CIGAR string limitations in the BAM format, necessitating the use of SAM or CRAM (https://github.com/samtools/hts-specs/issues/40). And, we were unable to complete an alignment of the ultra-long reads using BWA-MEM, and needed to adopt other algorithms, including GraphMap and NGM-LR, to align the reads. This required large amounts of compute time and RAM^37,38,50^. Availability of our data set has spurred the development of Minimap2 (ref. 39), and we recommend this long-read aligner for use in aligning ultra-long reads on a standard desktop computer.

Nanopore genotyping accuracy currently lags behind short-read sequencing instruments, due to a limited ability to discriminate between heterozygous and homozygous alleles, which arose from error rate and the depth of coverage in our sequencing data. We found that >99% of SNP calls were correct at homozygous reference sites, dropping to 91.4% at heterozygous and homozygous non-reference sites. Similarly, Nanopore and Illumina SV genotypes agreed at 81% of heterozygous and 90% of homozygous sites. These results highlight a need for structural variant genotyping tools for long, single-molecule sequencing reads. Using 1D^2^ chemistry (which sequences template and complement strands of the same molecule) or modeling nanopore ionic raw current, perhaps by incorporating training data from modified DNA, could potentially produce increased read accuracy. A complementary approach would be to increase coverage.

In summary, we provide evidence that a portable, biological nanopore sequencer could be used to sequence, assemble, and provisionally analyze structural variants and detect epigenetic marks, in point-of-care human genomics applications in the future.

## Methods

### Human DNA.

Human genomic DNA from the GM12878 human cell line (CEPH/Utah pedigree) was either purchased from Coriell as DNA (cat. no. NA12878) or extracted from the cultured cell line also purchased from Coriell (cat. no. GM12878). Cell culture was performed using Epstein–Barr virus (EBV)-transformed B lymphocyte culture from the GM12878 cell line in RPMI-1640 media with 2 mM L-glutamine and 15% FBS at 37 °C.

### QIAGEN DNA extraction.

DNA was extracted from cells using the QIAamp DNA mini kit (Qiagen). 5 × 10^6^ cells were spun at 300*g* for 5 min to pellet. The cells were resuspended in 200 μl PBS and DNA was extracted according to the manufacturer's instructions. DNA quality was assessed by running 1 μl on a genomic ScreenTape on the TapeStation 2200 (Agilent) to ensure a DNA Integrity Number (DIN) >7 (value for NA12878 was 9.3). Concentration of DNA was assessed using the dsDNA HS assay on a Qubit fluorometer (Thermo Fisher).

### Library preparation (SQK-LSK108 1D ligation genomic DNA).

1.5–2.5 μg human genomic DNA was sheared in a Covaris g-TUBE centrifuged at 5,000–6,000 r.p.m. in an Eppendorf 5424 (or equivalent) centrifuge for 2 × 1 min, inverting the tube between centrifugation steps.

DNA repair (NEBNext FFPE DNA Repair Mix, NEB M6630) was performed on purchased DNA but not on freshly extracted DNA. 8.5 μl nuclease-free water (NFW), 6.5 μl FFPE Repair Buffer and 2 μl FFPE DNA Repair Mix were added to the 46 μl sheared DNA. The mixture was incubated for 15 min at 20 °C, cleaned up using a 0.4× volume of AMPure XP beads (62 μl), incubated at room temperature with gentle mixing for 5 min, washed twice with 200 μl fresh 70% ethanol, pellet allowed to dry for 2 min, and DNA eluted in 46 μl NFW or EB (10 mM Tris pH 8.0). A 1 μl aliquot was quantified by fluorometry (Qubit) to ensure ≥1 μg DNA was retained.

End repair and dA-tailing (NEBNext Ultra II End-Repair/dA-tailing Module) was then performed by adding 7 μl Ultra II End-Prep buffer, 3 μl Ultra II End-Prep enzyme mix, and 5 μl NFW. The mixture was incubated at 20 °C for 10 min and 65 °C for 10 min. A 1× volume (60 μl) AMPure XP clean-up was performed and the DNA was eluted in 31 μl NFW. A 1-μl aliquot was quantified by fluorometry (Qubit) to ensure ≥700 ng DNA was retained.

Ligation was then performed by adding 20 μl Adaptor Mix (SQK-LSK108 Ligation Sequencing Kit 1D, Oxford Nanopore Technologies (ONT)) and 50 μl NEB Blunt/TA Master Mix (NEB, cat. no. M0367) to the 30 μl dA-tailed DNA, mixing gently and incubating at room temperature for 10 min.

The adaptor-ligated DNA was cleaned up by adding a 0.4 × volume (40 μl) of AMPure XP beads, incubating for 5 min at room temperature and resuspending the pellet twice in 140 μl ABB (SQK-LSK108). The purified-ligated DNA was resuspended by adding 25 μl ELB (SQK-LSK108) and resuspending the beads, incubating at room temperature for 10 min, pelleting the beads again, and transferring the supernatant (pre-sequencing mix or PSM) to a new tube. A 1-μl aliquot was quantified by fluorometry (Qubit) to ensure ≥500 ng DNA was retained.

### Sambrook and Russell DNA extraction.

This protocol was modified from Chapter 6 protocol 1 of Sambrook and Russell^51^. 5 × 10^7^ cells were spun at 4500*g* for 10 min to pellet. The cells were resuspended by pipette mixing in 100 μl PBS. 10 ml TLB was added (10 mM Tris-Cl pH 8.0, 25 mM EDTA pH 8.0, 0.5% (w/v) SDS, 20 μg/ml Qiagen RNase A), vortexed at full speed for 5 s and incubated at 37 °C for 1 h. 50 μl Proteinase K (Qiagen) was added and mixed by slow inversion ten times followed by 3 h at 50 °C with gentle mixing every 1 h. The lysate was phenol-purified using 10 ml buffer saturated phenol using phase-lock gel falcon tubes, followed by phenol:chloroform (1:1). The DNA was precipitated by the addition of 4 ml 5 M ammonium acetate and 30 ml ice-cold ethanol. DNA was recovered with a glass hook followed by washing twice in 70% ethanol. After spinning down at 10,000*g*, ethanol was removed followed by 10 min drying at 40 °C. 150 μl EB (Elution Buffer) was added to the DNA and left at 4 °C overnight to resuspend.

### Library preparation (SQK-RAD002 genomic DNA).

To obtain ultra-long reads, the standard Rapid Adapters (RAD002) protocol (SQK-RAD002 Rapid Sequencing Kit, ONT) for genomic DNA was modified as follows. 16 μl of DNA from the Sambrook extraction at approximately 1 μg/μl, manipulated with a cut-off P20 pipette tip, was placed in a 0.2 ml PCR tube, with 1 μl removed to confirm quantification value. 5 μl FRM was added and mixed slowly ten times by gentle pipetting with a cut-off pipette tip moving only 12 μl. After mixing, the sample was incubated at 30 °C for 1 min followed by 75 °C for 1 min on a thermocycler. After this, 1 μl RAD and 1 μl Blunt/TA ligase was added with slow mixing by pipetting using a cut-off tip moving only 14 μl ten times. The library was then incubated at room temperature for 30 min to allow ligation of RAD. To load the library, 25.5 μl RBF (Running Buffer with Fuel mix) was mixed with 27.5 μl NFW, and this was added to the library. Using a P100 cut-off tip set to 75 μl, this library was mixed by pipetting slowly five times. This extremely viscous sample was loaded onto the “spot on” port and entered the flow cell by capillary action. The standard loading beads were omitted from this protocol owing to excessive clumping when mixed with the viscous library.

### MinION sequencing.

MinION sequencing was performed as per manufacturer's guidelines using R9/R9.4 flow cells (FLO-MIN105/FLO-MIN106, ONT). MinION sequencing was controlled using Oxford Nanopore Technologies MinKNOW software. The specific versions of the software used varied from run to run but can be determined by inspection of fast5 files from the data set. Reads from all sites were copied off to a volume mounted on a CLIMB virtual server (http://www.climb.ac.uk) where metadata was extracted using poredb (https://github.com/nickloman/poredb) and base-calling performed using Metrichor (predominantly workflow ID 1200, although previous versions were used early on in the project) (http://www.metrichor.com). We note that base-calling in Metrichor has now been superseded by Albacore and is no longer available. Scrappie (https://github.com/nanoporetech/scrappie) was used for the chr20 comparisons using reads previously identified as being from this chromosome after mapping the Metrichor reads. Albacore 0.8.4 (available from the Oxford Nanopore Technologies user community) was used for the ultra-long read set, as this software became the recommended base-caller for nanopore reads in March 2017. Given the rapid development of upgrades to base-caller software we expect to periodically re-base-call these data and make the latest results available to the community through the Amazon Open Data site.

### Modified MinION running scripts.

In a number of instances, MinION sequencing control was shifted to customized MinKNOW scripts. These scripts provided enhanced pore utilization/data yields during sequencing, and operated by monitoring and adjusting flow cell bias-voltage (–180 mV to –250 mV), and used an event-yield-dependent (70% of initial hour in each segment) initiation of active pore channel assignment via remuxing (reselection of ideal pores for sequencing from each group of four wells available around each channel on the flowcell). More detailed information on these scripts can be found on the Oxford Nanopore Technologies user community. In addition, a patch for all files required to modify MinION running scripts compatible with MinKNOW 1.3.23 only is available (Supplementary Code 1).

### Live run monitoring.

To assist in choosing when to switch from a standard run script to a modified run protocol, a subset of runs was monitored with the assistance of the minControl tool, an alpha component of the minoTour suite of MinION run and analysis tools (https://github.com/minoTour/minoTour). minControl collects metrics about a run directly from the grouper software, which runs behind the standard ONT MinKNOW interface. minControl provides a historical log of yield measured in events from a flow cell enabling estimations of yield and the decay rate associated with loss of sequencing pores over time. MinKNOW yield is currently measured in events and is scaled by approximately 1.7 to estimate yield in bases.

### Assembly.

All “NG” statistics were computed using a genome size of 3,098,794,149 bp (3.1 Gbp), the size of GRCh38 excluding alt sites.

Canu v1.4 (+11 commits) r8006 (4a7090bd17c914f5c21bacbebf4add163e492d54) was used to assemble the initial 20-fold coverage data set:canu -p asm -d asm genomeSize=3.1g gridOptionsJobName=na12878nano ”gridOptions=–time 72:00:00–partition norm” -nanopore-raw rel2*.fastq.gz corMinCoverage=0 corMaxEvidenceErate=0.22 errorRate=0.045

These are the suggested low-coverage parameters from the Canu documentation, but with a decreased maximum evidence error rate. This specific parameter was decreased to reduced memory requirements after it was determined that the MinHash overlapping algorithm was underestimating error rates owing to systematic error in the reads. Counterintuitively, this systematic error makes two reads look more similar than they are, because they share more *k*-mers than expected under a random model. Manually decreasing the maximum overlap error rate threshold adjusted for this bias. The assembly took 40K CPU hours (25K to correct and 15K to assemble). This is about twofold slower than a comparable PacBio data set, mostly because of the higher noise and errors in the nanopore reads.

The same version of Canu was also used to assemble the 30-fold data set:canu -p asm -d asm genomeSize=3.1g gridOptionsJobName=na12878nano “gridOptions=–time 72:00:00–partition norm” -nanopore-raw rel3*.fastq.gz corMinCoverage=0 corMaxEvidenceErate=0.22 errorRate=0.045 ”corMhapOptions=–threshold 0.8–num-hashes 512–ordered-sketch-size 1000–ordered-kmer-size 14”

For this larger data set, overlapping was again tweaked by reducing the number of hashes used and increasing the minimum overlap identity threshold. This has the effect of lowering sensitivity to further compensate for the bias in the input reads. This assembly required 62K CPU hours (29K to correct, 33K to assemble) and a peak of 120 Gbp of memory, which is about fourfold slower than a comparable PacBio data set. The assembly ran on a cluster comprised of a mix of 48-thread dual-socket Intel E5-2680 v3 @ 2.50GHz CPUs with 128 Gbp of memory and 8-thread dual-socket Intel CPU E5-2698 v4 @ 2.20GHz CPUs with 1,024 Gbp of memory.

The combined data set incorporating an additional 5× coverage of ultra-long reads was assembled with an updated version of Canu v1.4 (+125 commits) r8120:canu -p asm -d asm genomeSize=3.1g gridOptionsJobName=na12878nano ”gridOptions=–time 72:00:00–partition norm” -nanopore-raw rel3*.fastq.gz -nanopore-raw rel4*.fastq.gz ”corMhapOptions=–threshold 0.8–num-hashes 512–ordered-sketch-size 1000–ordered-kmer-size 14” batOptions=”-dg 3 -db 3 -dr 1 -el 2000 -nofilter suspicious-lopsided”

This assembly required 151K CPU hours (15K to correct, 86K to trim, and 50K to assemble) and a peak of 112 Gbp of memory. These high runtimes are a consequence of the ultra-long reads. In particular, the current Canu trimming algorithm was not designed for reads of this extreme length and high error rate after correction and the algorithms used are not optimal.

### Assembly contiguity modeling.

Expected assembly contiguity was modeled on repeat tracks downloaded from the UCSC genome browser (http://hgdownload.soe.ucsc.edu/goldenPath/hg38/database/).

For a given repeat identity (0%, 90%, 95%, 98%, 99%, and 99.5%), all repeats with a lower identity estimate (genomicSuperDups and chainSelf) were filtered and overlapping repeats were merged. Gaps in the reference were also considered as repeats. To compute the maximum repeat length likely to be spanned by a given sequence distribution, the probability of an unspanned repeat of a fixed length was estimated for all lengths between 1 and 100 kbp in steps of 1 kbp using an equation from http://data-science-sequencing.github.io/lectures/lecture7/^52,53,54^:

where *G* is the genome size, *L* is the read length, *a*_*i*_ is the number of repeats of length 1 ≤ *i* ≤ *L* − 2, *N* is the number of reads ≥ *L*, and *c* is the coverage in reads ≥ *L*. We used the distribution of all repeats for *a*_*i*_ and plotted the shortest repeat length such that *P* (*at least one repeat is unbridged*) > 0.05 for real sequencing length distributions both nanopore and PacBio sequencing runs. Assemblies of the data were plotted at their predicted spanned read length on the *x* axis and NG50 on the *y* axis for comparison with the model. A 30× run of ultra-long coverage was simulated from the 5× dataset by repeating each ultra-long read six times.

### Assembly validation and structural variant analysis.

Assemblies were aligned using MUMmer v3.23 with parameters “-l 20 -c 500 -maxmatch” for the raw assemblies and “-l 100 -c 500 -maxmatch” for the polished assemblies. Output was processed with dnadiff to report average 1-to-1 alignment identity. The MUMmer coords file was converted to a tiling using the scripts from Berlin *et al*.^55^ with the command:python convertToTiling.py 10000 90 100000

and drawn using the coloredChromosomes package^56^. Since the reference is a composite of human genomes and there are true variations between the reference and NA12878, we also computed a reference-free estimate of identity. A 30-fold subset of the Genome In a Bottle Illumina data set for NA12878 (ref. 20) was downloaded from ftp://ftp-trace.ncbi.nlm.nih.gov/giab/ftp/data/NA12878/NIST_NA12878_HG001_HiSeq_300x/RMNISTHS_30xdownsample.bam. Samtools fastq was used to extract fastq paired-end data for the full data set and for the reads mapping to chromosome 20. The reads were aligned to the whole genome assembly and chromosome 20 assemblies with BWA-MEM 0.7.12-r1039. BWA-MEM is a component of the BWA package and was chosen because of its speed and ubiquitous use in sequence mapping and analysis pipelines. Aside from the difficulties of mapping the ultra-long reads unique to this work, any other mapper could be used instead. Variants were identified using FreeBayes v1.0.2 (ref. 57), a widely used method originally developed for short-read sequencing but also applicable to long reads, with the command:freebayes -C 2 -0 -O -q 20 -z 0.10 -E 0 -X -u -p 2 -F 0.6 -b alignments.bam -v asm.bayes.vcf -f asm.fasta

The length of all variants was summed and the total number of bases with at least 3× coverage was summed using samtools depth. QV was computed as  and identity was computed as  Dotplots were generated with “mummerplot–fat” using the 1-to-1 filtered matches.

A previously published GM12878 PacBio assembly^5^ was aligned as above with MUMmer v3.23. The resulting alignment files were uploaded to Assemblytics^58^ to identify structural variants and generate summary figures. Versus GRCh38, the PacBio assembly identified 10,747 structural variants affecting 10.84 Mbp, and reported an equal balance of insertions and deletions (2,361 vs. 2,724), with a peak at approximately 300 bp corresponding to Alu repeats (Supplementary Fig. 5a and Supplementary Table 6). The high error rate of the nanopore assembly resulted in a much larger number of identified variants (69,151) affecting 23.45 Mbp, with a strong deletion bias (3,900 insertions vs. 28,791 deletions) (Supplementary Fig. 5b and Supplementary Table 6). The Illumina-polished assembly reduced the total variants (47,073) affecting 16.24 Mbp but the deletion bias persisted (2,840 insertions vs. 20,797 deletions) (Supplementary Fig. 5c and Supplementary Table 6).

### Base-call analysis.

Sequences were aligned to the 1000 Genome GRCh38 reference (ftp://ftp.1000genomes.ebi.ac.uk/vol1/ftp/technical/reference/GRCh38_reference_genome/GRCh38_full_analysis_set_plus_decoy_hla.fa.sa) using BWA-MEM version 0.7.12-r1039 with the “-x ont2d” option^59^. The BAM alignments were converted to PAF format^60^ and CIGAR-strings parsed to convert alignments to an identity. Summary statistics for each flow cell were tabulated separately and combined. Alignment length versus identity was plotted using smoothScatter in R. Depth of coverage statistics for each flow cell were obtained from “samtools depth -a” and combined. As for the assembly statistics, a genome size of 3,098,794,149 bp was used to compute bases covered. The mean coverage was 25.63 (63.20 s.d.). The minimum coverage was 0 and the maximum was 44,391. Excluding 0-coverage regions, the mean coverage was 27.41 (64.98 s.d.). The coverage histogram was plotted compared with randomly generated Poisson values generated with R's rpois function with ë = 27.4074.

Metrichor reads mapping to human chromosome 20 were additionally base-called with Scrappie v0.2.7. Scrappie reads composed primarily of low-complexity sequence were identified using the sdust program included with Minimap (commit: 17d5bd12290e0e8a48a5df5afaeaef4d171aa133)^60^ with default parameters (-w 64 -t 20). The total length of the windows in a single sequence were merged and divided by read length to compute percentage of low-complexity sequence in each read. Any read for which this percentage exceeded 50% was removed from downstream analysis. Without this filtering, BWA-MEM did not complete mapping the sequences after >30 days of runtime on 16-cores. Similar filtering on the Metrichor-based reads had only a limited effect on the data set.

To measure homopolymer accuracy, we extracted pairwise read-to-reference alignments for reads spanning all homopolymers of length 2 or greater. For efficiency, at most 1,000 randomly selected instances were considered for each homopolymer length. Each homopolymer so identified is enclosed by two non-homopolymer “boundary” bases (e.g., the T and G in TAAAG). The number of match, mismatch, insertion, and deletion alignment operations between the boundary bases was tabulated for each homopolymer, and alignments not anchored at the boundary bases with match/mismatch operations were ignored. Homopolymer call length was reported as the number of inserted bases minus the number of deleted bases in the extracted alignment, quantifying the difference between expected and observed sequence length. All base callers with the exception of Scrappie failed in large homopolymer stretches (e.g., Supplementary Fig. 3), consistently capping homopolymers at 5 bp (the *k*-mer length of the model). Scrappie shows significant improvement, but tended to slightly overcall short homopolymers and undercall longer ones (Fig. 2b).

To quantify deviations from the expected 50:50 allele ratio at heterozygous sites, 25,541 homozygous and 46,098 heterozygous SNP positions on chromosome 20 were extracted from the Illumina Platinum Genomes project VCF for GM12878, requiring a minimum distance of 10 bp between SNP positions. Scrappie base calls at these positions were extracted using samtools mpileup. Deviation from the expected allelic ratio was defined as *d* = abs(0.5 – [allele A coverage]/[allele A coverage + allele B coverage]). Averaged over all evaluated heterozygous SNPs, *d* = 0.13 and 90% of SNPs have *d* ≤ 0.27 (corresponding to approximately ≥25% coverage on the minor allele). Results were similar when stratified by SNP type.

### Assembly polishing with nanopolish.

We ran the nanopolish consensus-calling algorithm^14^ on the chromosome 20 assemblies described above. For each assembly we sampled candidate variants from the base-called reads used to construct the contigs (using the “–alternative-basecalls” option) and input the original fast5 files (generated by the base-caller in the Metrichor computing platform) into a hidden Markov model, as these files contained the annotated events that the HMM relies on. The reads were mapped to the draft assembly using BWA-MEM with the “-x ont2d” option.

Each assembly was polished in 50,000-bp segments, and the individual segments were merged into the final consensus. The nanopolish jobs were run using default parameters except the “–fix-homopolymers” and “–min-candidate-frequency 0.01” options were applied.

### Assembly annotation.

Comparative Annotation Toolkit (CAT) (https://github.com/ComparativeGenomicsToolkit/Comparative-Annotation-Toolkit/commit/c9503e7ad7718a935b10a72f75302caa5accb15e) was run on both the polished and unpolished assemblies. CAT uses whole genome alignments to project transcripts from a high-quality reference genome to other genomes in the alignment^61^. The gene finding tool AUGUSTUS is used to clean up these transcript projections and a combined gene set is generated^62^.

To guide the annotation process, we obtained human RNA-seq data from SRA for a variety of tissues (Supplementary Table 7) and aligned them to both GRCh38 and the two assembly versions. GENCODE V24 was used as the reference annotation. Two separate progressiveCactus^63^ alignments were generated for each assembly version with the chimpanzee genome as an outgroup.

The frequency of frameshifting insertions or deletions (indels) in transcripts was evaluated by performing pairwise CDS (coding DNA sequence) sequence alignments using BLAT in a translated protein parameterization. Alignments were performed both on raw transMap output as well as on the final consensus transcripts.

Paralogous alignments of a source transcript were resolved through a heuristic combination of alignment coverage, identity, and synteny. Synteny is measured by counting how many gene projections near the current projection match the reference genome. In the case where multiple isoforms of a gene end up in different loci as the result of this process, a rescuing process is performed that chooses the highest scoring locus to place all isoforms at so that isoforms do not end up on different contigs. Through this process, a 1-1 orthology relationship is defined.

### MHC analysis.

The ultra-long assembly contains the MHC region between positions 2–6 Mb within a single 16-Mbp contig (tig01415017). Heterozygous sites were extracted by mapping Illumina reads to the polished assembly using BWA-MEM with default parameters. Alignments were post-processed according to the GATK 3.7 whole-genome variant calling pipeline, except for the “-T IndelRealigner” step using “–consensusDeterminationModel USE_READS”. The -T HaplotypeCaller parameter was used for variant calling. WhatsHap^64^ was used to phase the Illumina variants with Nanopore reads reported to be contained in the contig by Canu. WhatsHap was modified to accept CRAM (http://genome.cshlp.org/content/21/5/734.long, https://bitbucket.org/skoren/whatshap) output since BAM files could not represent long CIGAR strings at the time of this analysis (https://github.com/samtools/hts-specs/issues/40). First, WhatsHap was run excluding any ultra-long sequences. This generated 18 phase blocks across the MHC. When ultra-long sequences were included the result was a single phase block comprising the entire MHC, supporting the utility of ultra-long reads in resolving haplotypes across large, complex regions in the genome. Nanopore reads were aligned back to the assembly using NGM-LR (CoNvex Gap-cost alignMents for Long Reads)^38^ and the combined VCF file used for phasing. Reads with more than one phasing marker were classified as haplotype A or B when >55% of their variants were in agreement (Fig. 5a). A new assembly was generated for haplotypes A and B using only reads assigned to each haplotype as well as reads marked homozygous. The assemblies were polished by Pilon 1.21(ref. 26) using the SGE pipeline at https://github.com/skoren/PilonGrid. Pilon was given all reads mapping to the MHC.

Exon sequences belonging to the six classical HLA genes were extracted from the phased assembly, and HLA types called at G group resolution. These results were compared to GM12878 HLA type reference data. For the class I and II HLA genes, with the exception of one DRB1 haplotype, there was good agreement between the best-matching reference type and the alleles called from the assembly (edit distance 0–1). Detailed examination of HLA-DRB1, however, showed that one exon (exon 2) is different from all reference types in the assembly, a likely error in the assembly sequence.

GM12878 G group HLA types for HLA-A/B/C, HLA-DQA1, HLA-DQB1, and HLA-DRB1 are from ref. 65; the presence of exactly one HLA-DRB3 allele is expected due to linkage with HLA-DRB1 (DRB1*03 is associated with HLA-DRB3, and DRB1*01 has no DRB3/4/5 association).

### Genotyping SNPs using nanopolish.

Nanopolish was used for genotyping the subset of reads that mapped to human chromosome 20. The 1000 Genomes phase 3 variant set for GRCh38 was used as a reference and filtered to include only chromosome 20 SNPs that were not singletons (Allele Count ≥ 2). This set of SNPs was input into “nanopolish variants” in genotyping mode (“–genotype”). The genotyping method extends the variant calling framework previously described^12^ to consider pairs of haplotypes, allowing it to be applied to diploid genomes (option “–ploidy 2”). To evaluate their accuracy, genotype calls were compared to the “platinum calls” generated by Illumina^23^. When evaluating the correctness of a nanopore call, we required the log-likelihood ratio of a variant call (heterozygous or homozygous non-reference) to be at least 30, otherwise, we considered the site to be homozygous reference.

### Estimating SV genotyping sensitivity.

Previously identified high-confidence GM12878 SVs, validated with Moleculo and/or PacBio long reads, were used to determine genotyping sensitivity^29^. Using LUMPY^28^, we recalled SVs in the Platinum Genomes NA12878 Illumina data set (paired-end reads; European Nucleotide Archive, Run Accession ERR194147), intersected these calls with the aforementioned high confidence set, and genotyped the resulting calls using SVTyper^28^ and the same Platinum alignments, generating a set of 2,414 high-confidence duplications and deletions with accompanying genotypes. Nanopore reads from all flow cells were mapped using BWA-MEM (bwa mem -k15 -W30 -r10 -B2 -O2 -L0), and then merged into release-specific BAM files. Merged BAM files were subsampled using Samtools (samtools view -s $COVERAGE_FRACTION) to approximate coverage values as shown in Figure 2a. SVs were then genotyped in each subsampled BAM file using a modified version of SVTyper (http://github.com/tomsasani/svtyper). Generally, long nanopore reads are subject to higher rates of mismatches, insertions, and deletions than short Illumina reads. These features can result in 'bleed-through' alignments, where reads align past the true breakpoint of an SV^66^. The modifications to SVTyper attempt to correct for the bleed-through phenomenon by allowing reads to align past the breakpoint, yet still support an alternate genotype. All modifications to SVTyper are documented in the source code available at the GitHub repository listed above (commit ID: d70de9c) (Supplementary Code 2). Nanopore- and Illumina-derived genotypes were then compared as a function of subsampled nanopore sequencing coverage.

The false-discovery rate of our SVTyper genotyping strategy was estimated by randomly permuting the genomic locations of the original SVs using BEDTools “shuffle”^67^. Centromeric, telomeric, and “gap” regions (as defined by the UCSC Genome Browser) were excluded when assigning randomly selected breakpoints to each SV. The randomly shuffled SVs were then genotyped in Illumina and nanopore data in the same manner as before. It is expected that the alignments at shuffled SV intervals would almost always support a homozygous reference genotype. So, all instances in which Illumina data supported a homozygous reference genotype, yet the nanopore data called a non-homozygous reference genotype, were considered false positives. SV coordinates were shuffled and genotyped 1,000 times and the average false-discovery rate over all iterations was 6.4%.

Nanopore and PacBio genotyping sensitivity was compared to a subset of our high-confidence SV set. Because our high-confidence set includes only “DUP” and “DEL” variants, and the Genome in a Bottle (GIAB) PacBio SV VCF (ftp://ftp-trace.ncbi.nlm.nih.gov/giab/ftp/data/NA12878/NA12878_PacBio_MtSinai/NA12878.sorted.vcf.gz) does not report “DUP” variants, we compared genotypes at deletions with genomic coordinates that shared reciprocal overlap of at least 0.5 between the GIAB VCF and our high-confidence SV VCF. We then compared nanopore genotypes (as determined by SVTyper) with the genotypes reported in the GIAB SV VCF. Importantly, the GIAB VCF was derived from a ∼44× coverage data set, whereas our data set (containing data from both releases) represents only about ∼32× coverage of the genome. Additionally, all nanopore data used in this analysis were aligned using BWA, while GIAB PacBio data were aligned using BLASR^68^.

### Scaling marginAlign and signalAlign data analysis pipelines.

To handle the large data volume, the original marginAlign and signalAlign algorithms were ported to cloud infrastructures using the Toil batch system^69^. Toil allows for computational resources to be scaled horizontally and vertically as a given experiment requires and enables researchers to perform their own experiments in identical conditions. All of the workflows used and the source code is freely available from https://github.com/ArtRand/toil-signalAlign and https://github.com/ArtRand/toil-marginAlign. Workflow diagrams are shown in Supplementary Figure 10.

### Generating a controlled set of methylated control DNA samples.

For signalAlign, DNA methylation control standards were obtained from Zymo Research (cat. no. D5013). The standards contain a whole-genome-amplified (WGA) DNA substrate that lacks methylation and a WGA DNA substrate that has been enzymatically treated so all CpG dinucleotides contain 5-methylcytosines. The two substrates were sequenced independently on two different flow cells using the sequencing protocol described above. Otherwise, training for signalAlign and nanopolish was carried out as previously described^35,36^.

### 5-methylcytosine detection with signalAlign.

The signalAlign algorithm uses a variable order hidden Markov model combined with a hierarchical Dirichlet process (HMM-HDP) to infer base modifications in a reference sequence using the ionic current signal produced by nanopore sequencing^70^. The ionic current signal is simultaneously influenced by multiple nucleotides as the strand passes through the nanopore. Correspondingly, signalAlign models each ionic current state as a nucleotide *k*-mer. The model allows a base in the reference sequence to have any of multiple methylation states (in this case 5-methy cytosine or canonical cytosine). The model ties the probabilities of consistently methylated *k*-mers by configuring the HMM in a variable order meta-structure that allows for multiple paths over a reference *k*-mer depending on the number of methylation possibilities. To learn the ionic current distributions for methylated *k*-mers, signalAlign estimates the posterior mean density for each *k*-mer's distribution of ionic currents using a Markov chain Monte Carlo (MCMC) algorithm given a set of *k*-mer-to-ionic current assignments. Using the full model, the posterior for each methylation status is calculated for all cytosines in CpG dinucleotides.

### 5-methylcytosine detection with nanopolish.

Previous work describes using nanopolish to call 5-methylcytosine in a CpG context using a hidden Markov model^36^. The output of the nanopolish calling procedure is a log-likelihood ratio, where a positive log-likelihood ratio indicates evidence for methylation. Nanopolish groups nearby CpG sites together and calls the group jointly, assigning the same methylation status to each site in the group. To allow comparison to the bisulfite data each such group was broken up into its constituent CpG sites, which all have the same methylation frequency. Percent-methylation was calculated by converting the log-likelihood ratio to a binary methylated/unmethylated call for each read, and calculating the fraction of reads classified as methylated. A filtered score was also computed by first filtering reads where the absolute value of the log-likelihood ratio was less than 2.5 to remove ambiguous reads.

### Life Sciences Reporting Summary.

Further information on experimental design is available in the Life Sciences Reporting Summary.

### Data availability.

Sequence data including raw signal files (FAST5), event-level data (FAST5), base-calls (FASTQ) and alignments (BAM) are available as an Amazon Web Services Open Data set for download from https://github.com/nanopore-wgs-consortium/NA12878. Nanopore raw signal files and the 35× assembly are additionally archived and available from the European Nucleotide Archive under accession PRJEB23027.

## Additional information

**Publisher's note:** Springer Nature remains neutral with regard to jurisdictional claims in published maps and institutional affiliations.

## Supplementary Information

### Integrated supplementary information

Supplementary Figure 1 Read Complexity A) Density plot showing the percentage of read length masked by the ‘dust’ program, which identifies low-complexity sequence (simple repeats). Scrappie outputs a significantly larger fraction of low-complexity bases, including some reads that are entirely low-complexity sequence.
B) Density plot showing the % identity for reads, weighted by alignment length, basecalled with Metrichor and Scrappie both pre and post correction.

Supplementary Figure 2 Basecall Bias Confusion matrices describing call bias for the base calling algorithms used from high-confidence alignments.

Supplementary Figure 3 Illustrative homopolymer resolution by basecaller IGV plot showing a poly-A region and aligned reads from the Metrichor and Scrappie base callers. The top two tracks show coverage across the region, and the bottom two tracks show the read alignments. Horizontal black bars in the read alignment tracks indicate deletions. Colorful bars indicate mismatches. Metrichor fails to call the homopolymer entirely, but Scrappie produces more reasonable calls across this region.

Supplementary Figure 4 Assembled contigs against reference A) Alignment dotplot of the nanopore GM12878 assembly aligned against human reference GRCh38 showing overall structural agreement. Human chromosomes are arranged along the x-axis with assembled contigs along the y-axis. Grid lines indicate chromosome and contig boundaries. Forward-strand matches are in red and reverse-complement in blue.
B) Chromosomes plot illustrating the contiguity of the 30× nanopore assembly. Contig NG50 was 3 Mbp. Contig and alignment boundaries, not cytogenetic bands, are represented by a color switch, so regions of continuous color indicate regions of continuous sequence. White areas indicate unmapped sequence, usually caused by N’s in the reference genome. The MHC region on chromosome 6 is labeled, which is reconstructed as described in the main text.

Supplementary Figure 5 Structural Variant Analysis Structural variants in the whole-genome nanopore assembly were identified using Assemblytics ^57^ and compared with a previous PacBio assembly ^5^. Histograms are given for insertion, deletion, repeat expansion/contraction, and tandem expansion/contraction SVs versus GRCh38. These are further broken into small (50–500 bp) and large (500–10000 bp) categories. Notably, the PacBio assembly shows a balanced rate of insertions and deletions, with a peak at 300 bp due to Alu insertion and deletion. In contrast, the nanopore assembly shows a strong deletion bias, with the majority of variants being deletions <500 bp. Note that this changes the y-axis scale and obscures the Alu peaks in these plots. Post-polishing, the deletion bias is reduced but is still significantly higher than PacBio. It is expected that assembly of Scrappie reads would further reduce the deletion bias observed.

Supplementary Figure 6 Assembly accuracy Accuracy of the 30× nanopore assembly before and after Illumina polishing. Modal accuracy of the nanopore-only assembly is ~96%. After Illumina polishing, this increases to >99%, with no substantial gain after 2 rounds of polishing.

Supplementary Figure 7 Sequences not found in the assembly Distribution of repeat classes observed in unassembled sequence reads and contigs that were not incorporated in primary assembly. Percentage of bases for each repeat class are listed for both unassembled reads and assembled, yet unplaced contigs. Proportion of repeat families within the general repeat class are provided using sequence annotation by RepeatMasker (RepBase22.03).

Supplementary Figure 8 Alignment artifacts complicate SV genotyping A) Illustrative IGV screenshot of an expected homozygous alternate deletion (interval displayed at bottom), with PacBio, nanopore, and Illumina alignments, as well as SINE and LINE tracks, displayed. PacBio reads appear to be spuriously aligned to a region containing a single LINE1 element, and were reported to support a heterozygous genotype in the GIAB SV VCF. A total of 44 SINE/LINE elements overlap SVs for which Illumina reads support a homozygous alternate genotype and the PacBio GIAB VCF reports a heterozygous genotype (requiring a reciprocal overlap of at least 0.75).
B) IGV screenshot of an expected heterozygous deletion (interval at bottom), with nanopore and Illumina alignments shown. A homopolymer run of thymines at the start of the deletion is not recovered by nanopore reads. Due to the resulting preponderance of indels, reads that would normally be classified as supporting a reference genotype are instead classified as alternate, and the heterozygous SV is genotyped as homozygous alternate in the nanopore alignments.

Supplementary Figure 9 Methylation A) Native DNA methylation detection on a selected portion of chromosome 20. Individual plots show 500 called cytosine bases ordered along chromosome 20. Total marginal probability of methylation is shown as black bar. High-confidence methylation calls from ENCODE (ENCSR890UQO), blue line, were filtered for positions where all reads called methylated or not methylation to remove ambiguity. Cytosine calls were filtered to only sites with coverage >= 10 reads in both data sets.
B) Receiver operating characteristic (ROC) plot describing SignalAlign as a binary classifier for individual 5-methyl cytosine detection (n=658,514)..

Supplementary Figure 10 marginAlign/SignalAlign Work Flow Workflow chart describing marginAlign and SignalAlign. All distributed steps were implemented as part of a Toil-pipeline to be run in the cloud. Dotted lines represent repeating steps (iterations). The pipeline was repeated twice to validate the result.

Supplementary Figure 11 Ultra-long reads DNA extraction Pulsed-field gel showing fragment sizes of; *E. coli* MG1655 DNA extracted using the Qiagen Genomic DNA buffer set and a Qiagen 500G column following the protocol for bacteria (lanes 2 and 3), E. coli MG1655 DNA extracted using the Sambrook and Russell phenol/chloroform protocol described in the methods section (lanes 4 and 5), E. coli MG1655 DNA extracted using a plug lysis method to preserve intact chromosomes (lanes 6 and 7) and Human NA12878 DNA extracted using the Sambrook and Russell phenol/chloroform protocol described in the methods section (lane 8 and 9). For each pair of samples one was irradiated with approximately 35 Gray ionising radiation to introduce double-strand breaks, this improves the intensity of the band representing the 4.6 Mb *E. coli* MG1655 chromosome. A 1.2% PFG agarose gel made with 0.5% TBE and run on a Bio-Rad CHEF Mapper at 14°C for 20 hours 46 minutes with a two-state 120° included angle, 6 V/cm gradient, initial switch time 0.64s and final switch time 1m 13.22s. The gel was ethidium bromide stained and imaged on a Bio-Rad Gel Doc XR system.

### Supplementary information

Supplementary Text and Figures Supplementary Figures 1–11 (PDF 2215 kb)

Life Sciences Reporting Summary (PDF 161 kb)

Supplementary Tables 1–4, 6–9, and 11 (PDF 630 kb)

Supplementary Table 5 Kmer Count Summaries Summary containing kmer counts with respect to chromosome 20 for each of the base callers used in this study (XLSX 161 kb)

Supplementary Table 10 Summary of gap closures within the canu reference assembly and ultra long reads from this study which span them. Chromosome GRCh38 (GCA_000001305.2) coordinates, chromosome banding information, and flanking assembled contigs are listed for each gapped region. A region is considered "closed" if it is spanned by our canuassembly and if the sequence structure observed at the assembly closure is supported by at least one 100 kb + read. The assembled contig ID from this study observed to close the gap is listed as Canu Assembly. The totalnumber of bases inserted from the gapped region are represented as Gap size (bp). New, inserted sequences are primarily repetitive (simple sequence repeats and transposable elements), as determined by RepeatMasker. Long reads that span the gaps and provide concordant evidence for the inserted sequence in the assembly are provided. Alignments of the long reads to the assembled contigs was performed using minimap2 37. (XLSX 53 kb)

Supplementary Code 1 Tuning Patches for MinKNOW 1.3.25 Oxford Nanopore scripts are considered confidential data. As such we release diffs to enable the recreation of the code used to control runs. Please note these scripts are only compatible with MinKNOW vs 1.3.25.Future updates to tuning scripts will be available through the Oxford Nanopore Community Forums. See the Instructions file for information on installing these scripts. (ZIP 8 kb)

Supplementary Code 2 SVTyper A version of SV-Typer modified for nanopore read profiles. The code is available at http://github.com/tomsasani/svtyper (commit ID: d70de9c). (ZIP 16 kb)

## Data Availability

European Nucleotide Archive
PRJEB23027 European Nucleotide Archive
ERR194147
