# Supplementary figures and images for: Nanopore sequencing and assembly of a human genome with ultra-long reads
Source: Nat Biotechnol. 2018 Jan 29;36(4):338–45. doi: 10.1038/nbt.4060 (PMC5889714; doi:10.1038/nbt.4060)

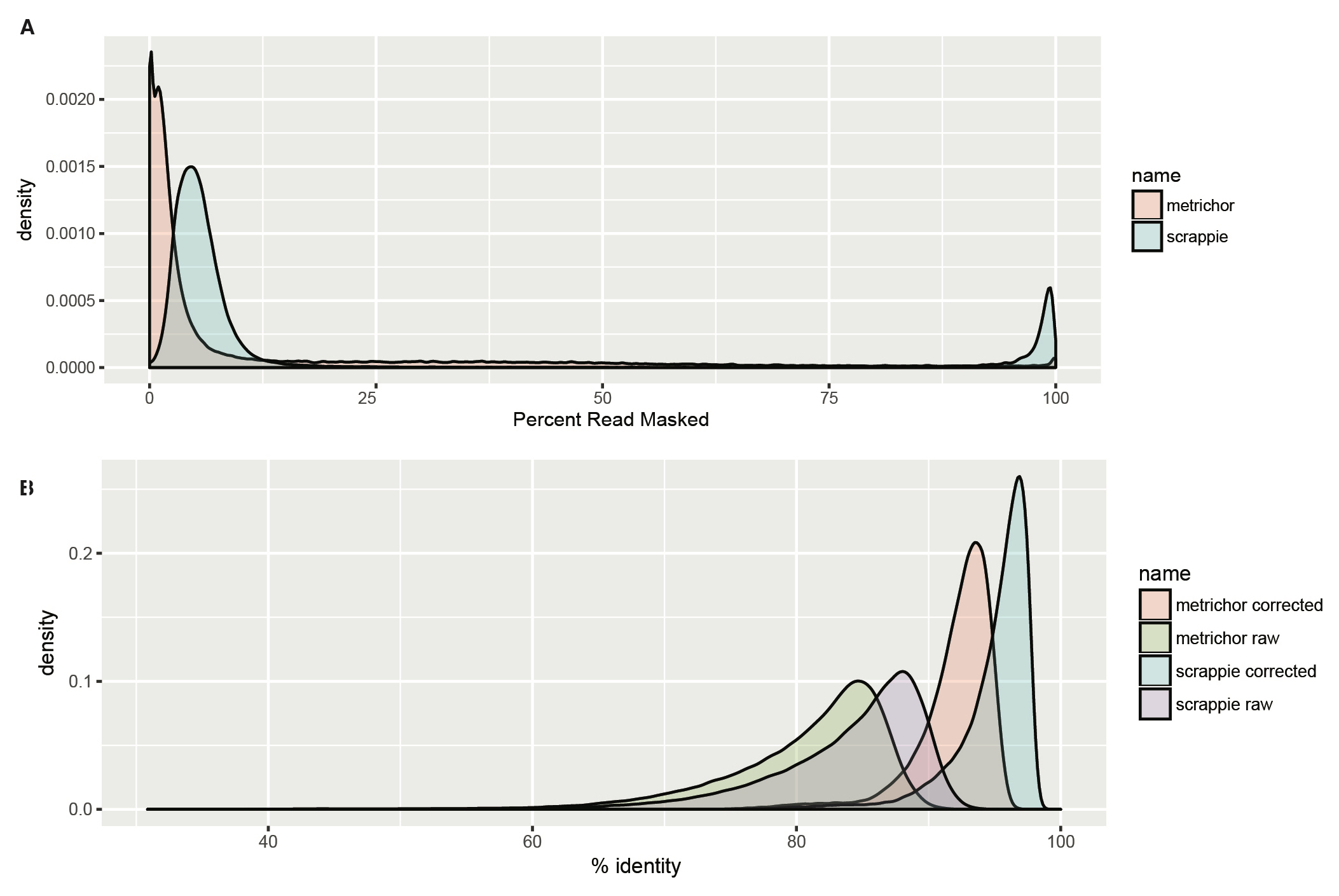

Supplement: Read Complexity — A) Density plot showing the percentage of read length masked by the ‘dust’ program, which identifies low-complexity sequence (simple repeats). Scrappie outputs a significantly larger fraction of low-complexity bases, including some reads that are entirely low-complexity sequence. B) Density plot showing the % identity for reads, weighted by alignment length, basecalled with Metrichor and Scrappie both pre and post correction. [file 41587_2018_Article_BFnbt4060_Fig6_ESM.jpg]

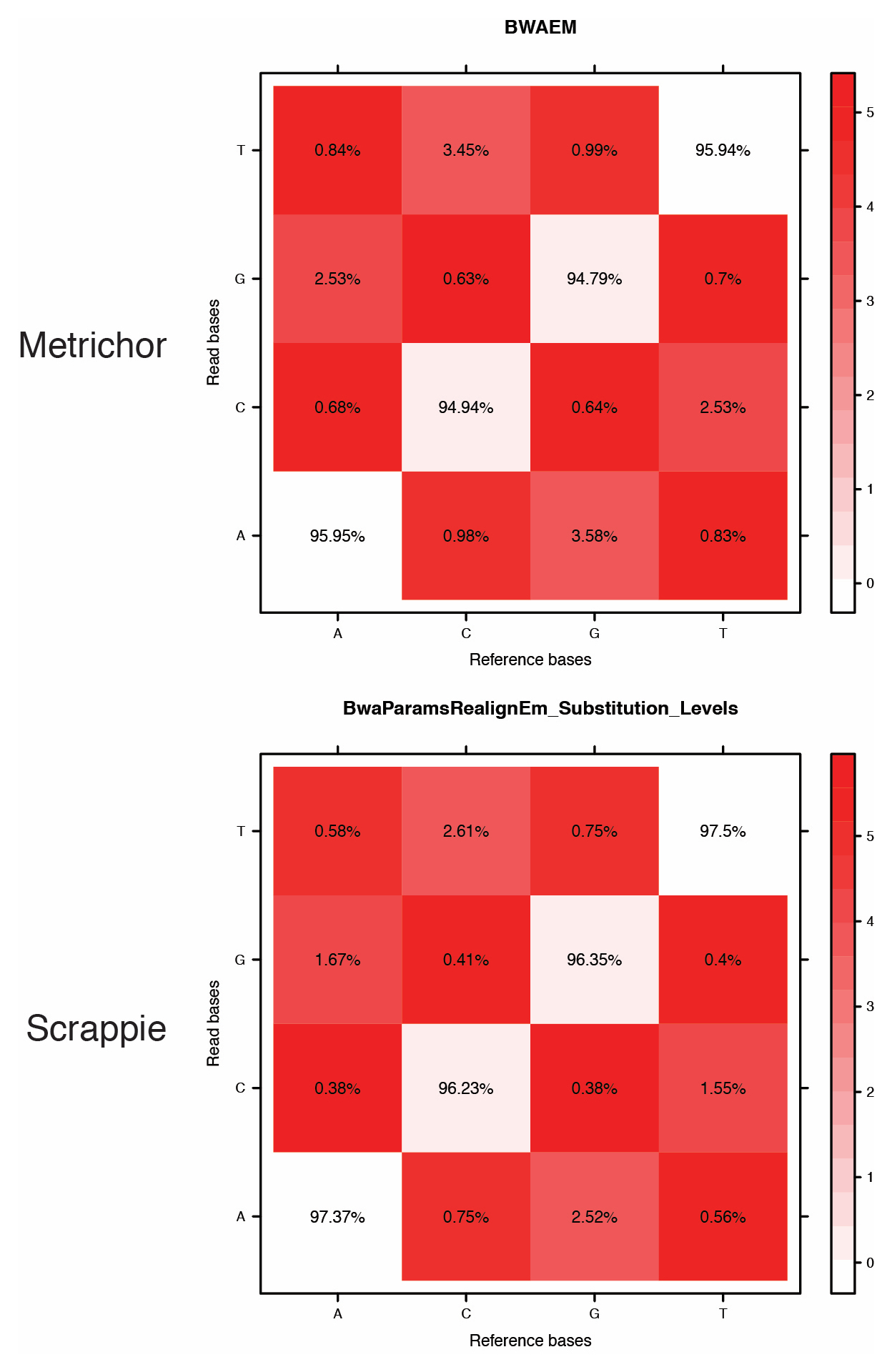

Supplement: Basecall Bias — Confusion matrices describing call bias for the base calling algorithms used from high-confidence alignments. [file 41587_2018_Article_BFnbt4060_Fig7_ESM.jpg]

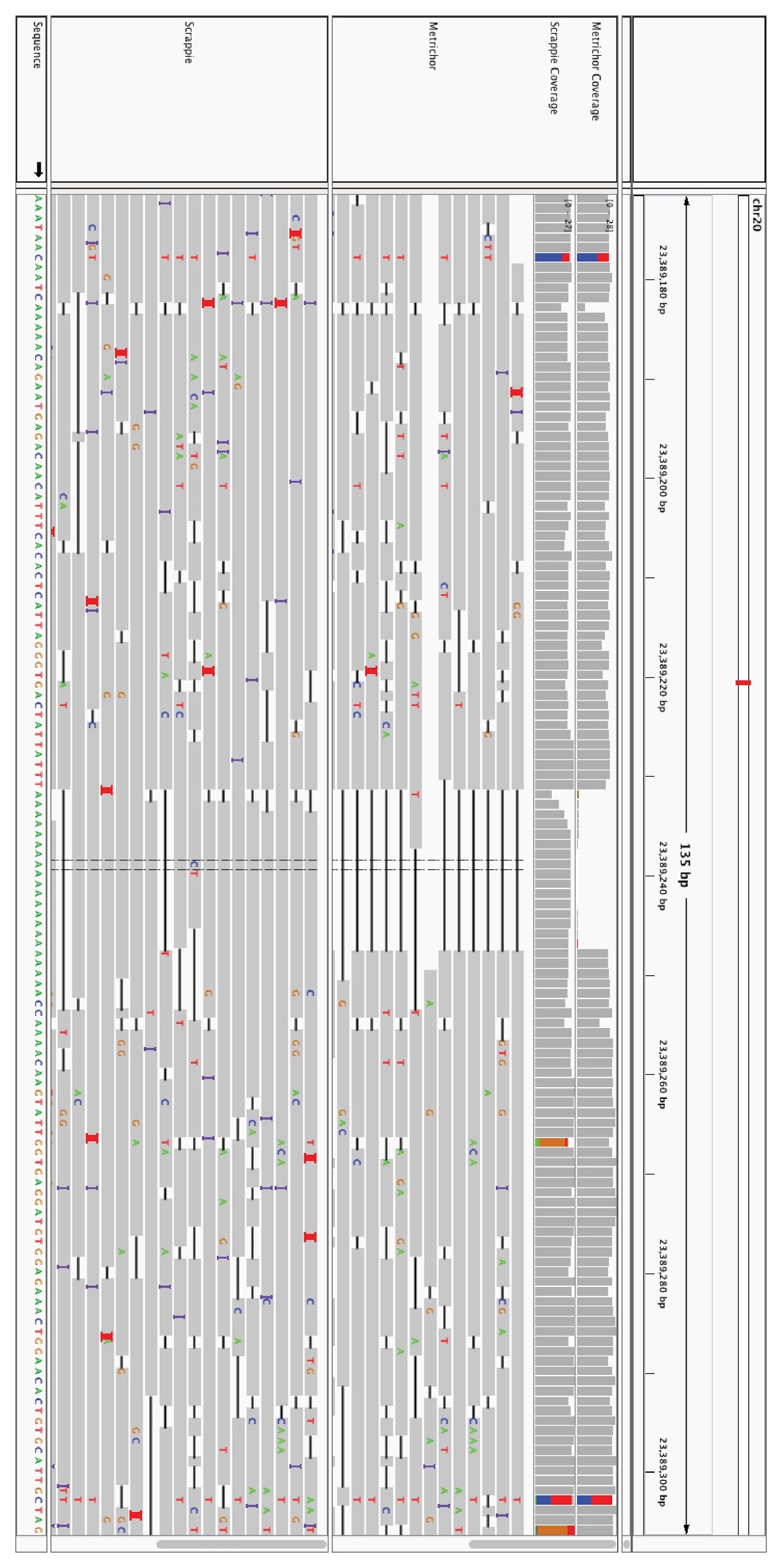

Supplement: Illustrative homopolymer resolution by basecaller — IGV plot showing a poly-A region and aligned reads from the Metrichor and Scrappie base callers. The top two tracks show coverage across the region, and the bottom two tracks show the read alignments. Horizontal black bars in the read alignment tracks indicate deletions. Colorful bars indicate mismatches. Metrichor fails to call the homopolymer entirely, but Scrappie produces more reasonable calls across this region. [file 41587_2018_Article_BFnbt4060_Fig8_ESM.jpg]

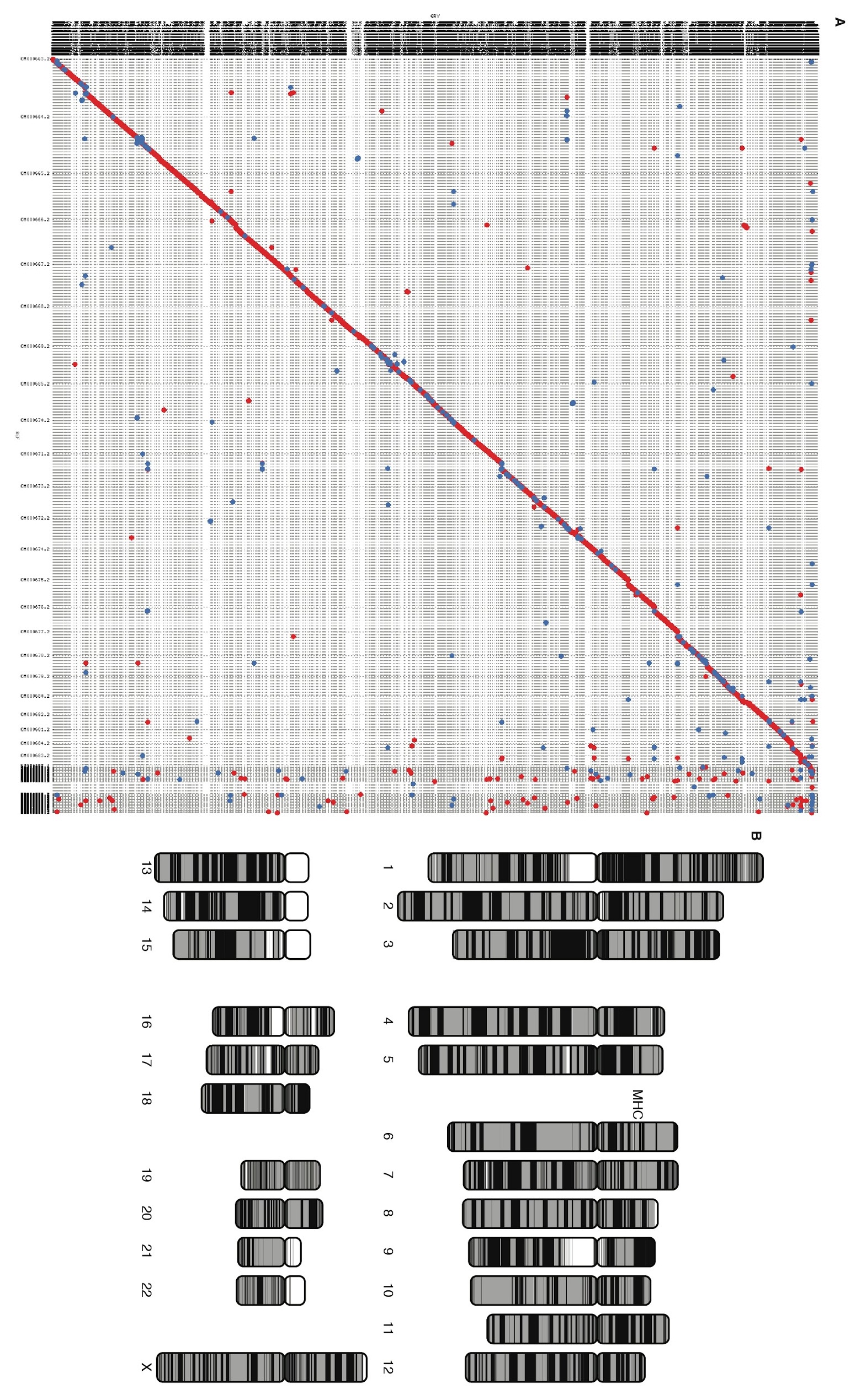

Supplement: Assembled contigs against reference — A) Alignment dotplot of the nanopore GM12878 assembly aligned against human reference GRCh38 showing overall structural agreement. Human chromosomes are arranged along the x-axis with assembled contigs along the y-axis. Grid lines indicate chromosome and contig boundaries. Forward-strand matches are in red and reverse-complement in blue. B) Chromosomes plot illustrating the contiguity of the 30× nanopore assembly. Contig NG50 was 3 Mbp. Contig and alignment boundaries, not cytogenetic bands, are represented by a color switch, so regions of continuous color indicate regions of continuous sequence. White areas indicate unmapped sequence, usually caused by N’s in the reference genome. The MHC region on chromosome 6 is labeled, which is reconstructed as described in the main text. [file 41587_2018_Article_BFnbt4060_Fig9_ESM.jpg]

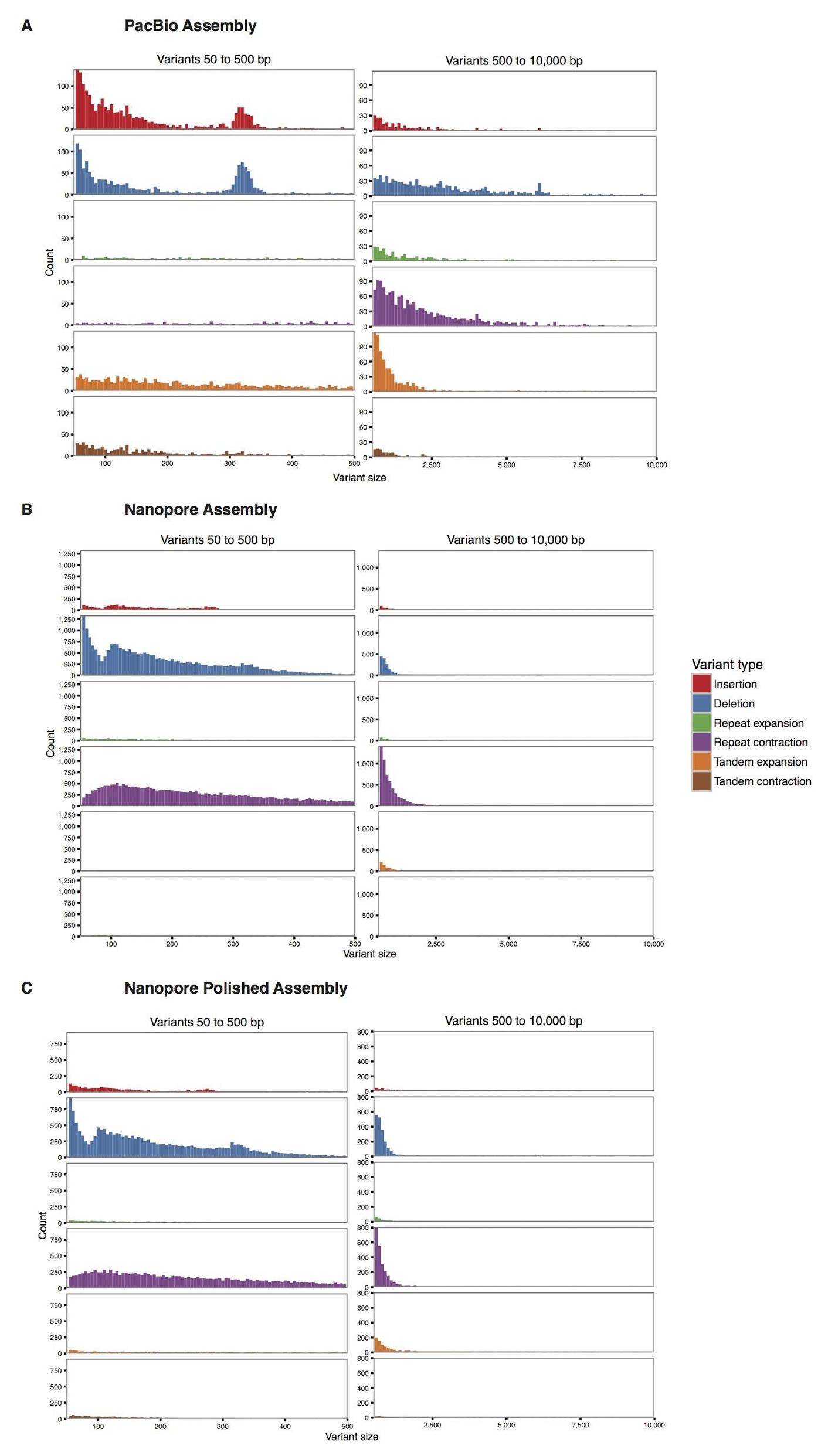

Supplement: Structural Variant Analysis — Structural variants in the whole-genome nanopore assembly were identified using Assemblytics 57 and compared with a previous PacBio assembly 5. Histograms are given for insertion, deletion, repeat expansion/contraction, and tandem expansion/contraction SVs versus GRCh38. These are further broken into small (50–500 bp) and large (500–10000 bp) categories. Notably, the PacBio assembly shows a balanced rate of insertions and deletions, with a peak at 300 bp due to Alu insertion and deletion. In contrast, the nanopore assembly shows a strong deletion bias, with the majority of variants being deletions <500 bp. Note that this changes the y-axis scale and obscures the Alu peaks in these plots. Post-polishing, the deletion bias is reduced but is still significantly higher than PacBio. It is expected that assembly of Scrappie reads would further reduce the deletion bias observed. [file 41587_2018_Article_BFnbt4060_Fig10_ESM.jpg]

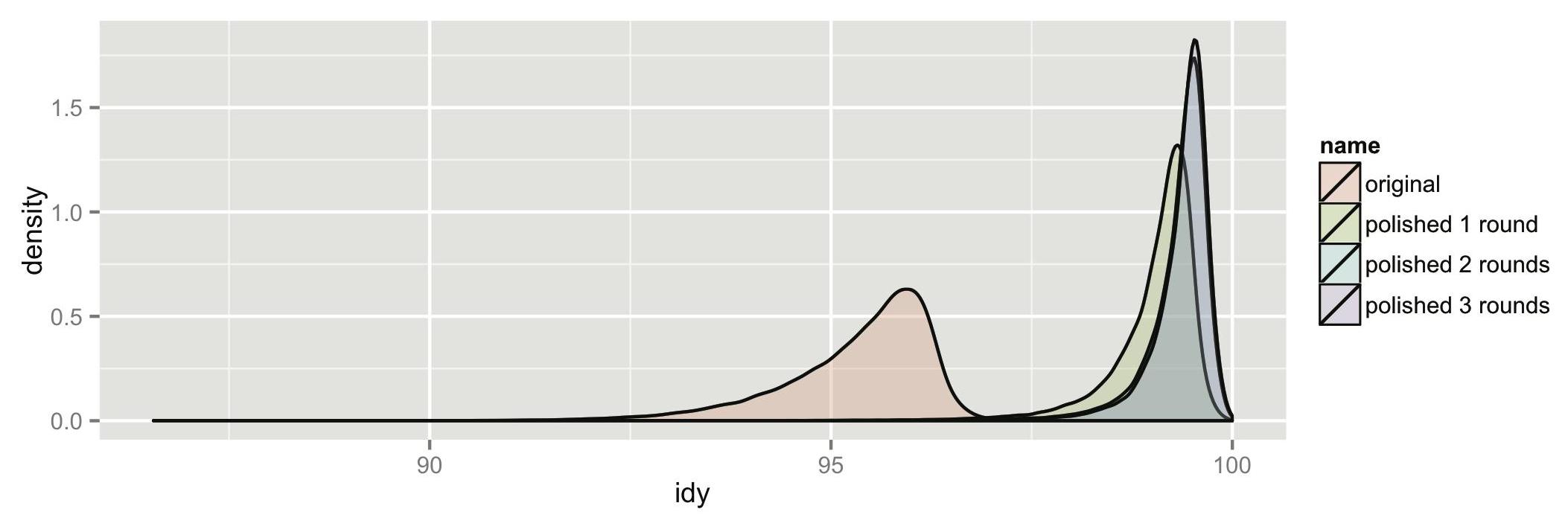

Supplement: Assembly accuracy — Accuracy of the 30× nanopore assembly before and after Illumina polishing. Modal accuracy of the nanopore-only assembly is ~96%. After Illumina polishing, this increases to >99%, with no substantial gain after 2 rounds of polishing. [file 41587_2018_Article_BFnbt4060_Fig11_ESM.jpg]

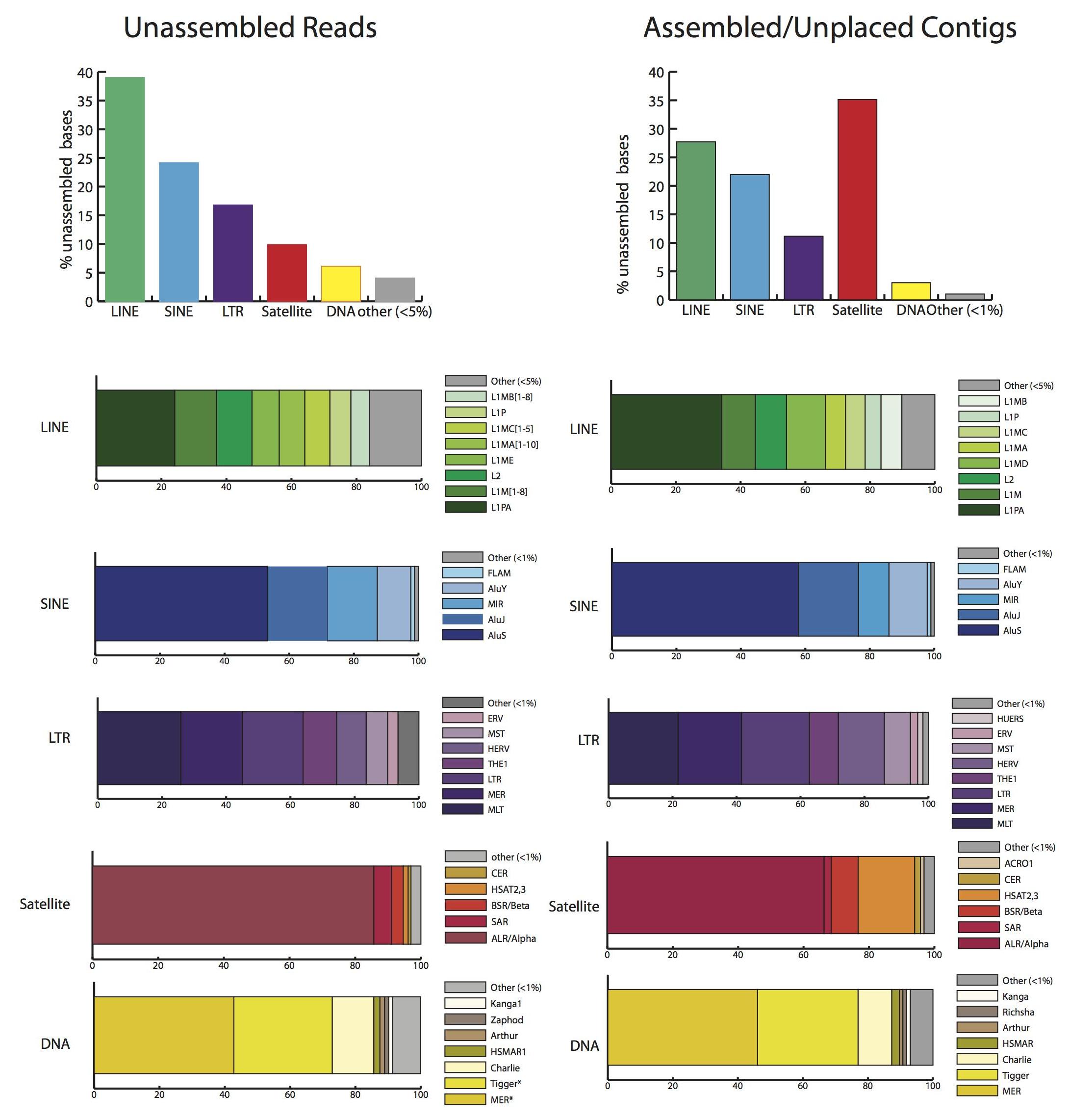

Supplement: Sequences not found in the assembly — Distribution of repeat classes observed in unassembled sequence reads and contigs that were not incorporated in primary assembly. Percentage of bases for each repeat class are listed for both unassembled reads and assembled, yet unplaced contigs. Proportion of repeat families within the general repeat class are provided using sequence annotation by RepeatMasker (RepBase22.03). [file 41587_2018_Article_BFnbt4060_Fig12_ESM.jpg]

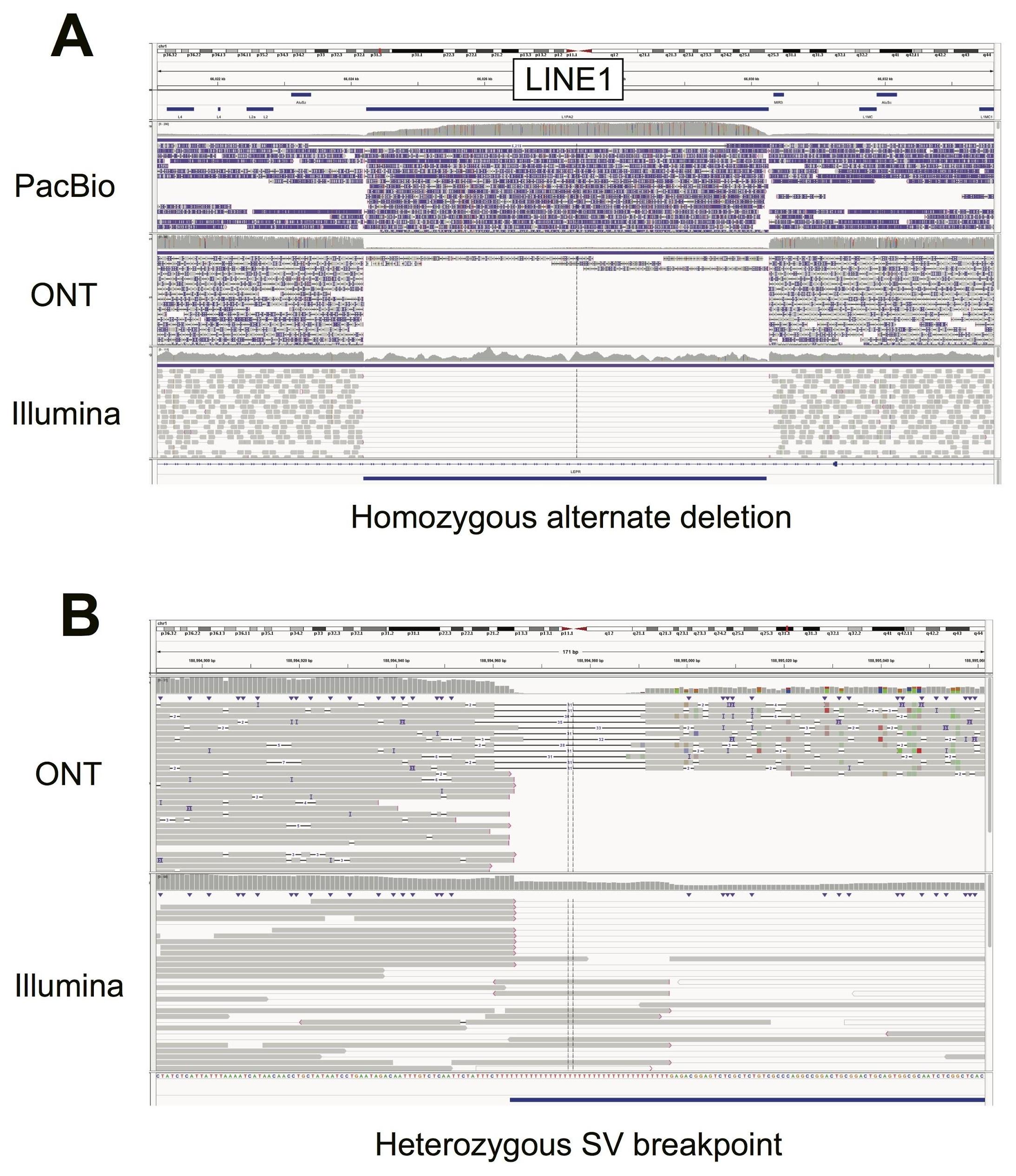

Supplement: Alignment artifacts complicate SV genotyping — A) Illustrative IGV screenshot of an expected homozygous alternate deletion (interval displayed at bottom), with PacBio, nanopore, and Illumina alignments, as well as SINE and LINE tracks, displayed. PacBio reads appear to be spuriously aligned to a region containing a single LINE1 element, and were reported to support a heterozygous genotype in the GIAB SV VCF. A total of 44 SINE/LINE elements overlap SVs for which Illumina reads support a homozygous alternate genotype and the PacBio GIAB VCF reports a heterozygous genotype (requiring a reciprocal overlap of at least 0.75). B) IGV screenshot of an expected heterozygous deletion (interval at bottom), with nanopore and Illumina alignments shown. A homopolymer run of thymines at the start of the deletion is not recovered by nanopore reads. Due to the resulting preponderance of indels, reads that would normally be classified as supporting a reference genotype are instead classified as alternate, and the heterozygous SV is genotyped as homozygous alternate in the nanopore alignments. [file 41587_2018_Article_BFnbt4060_Fig13_ESM.jpg]

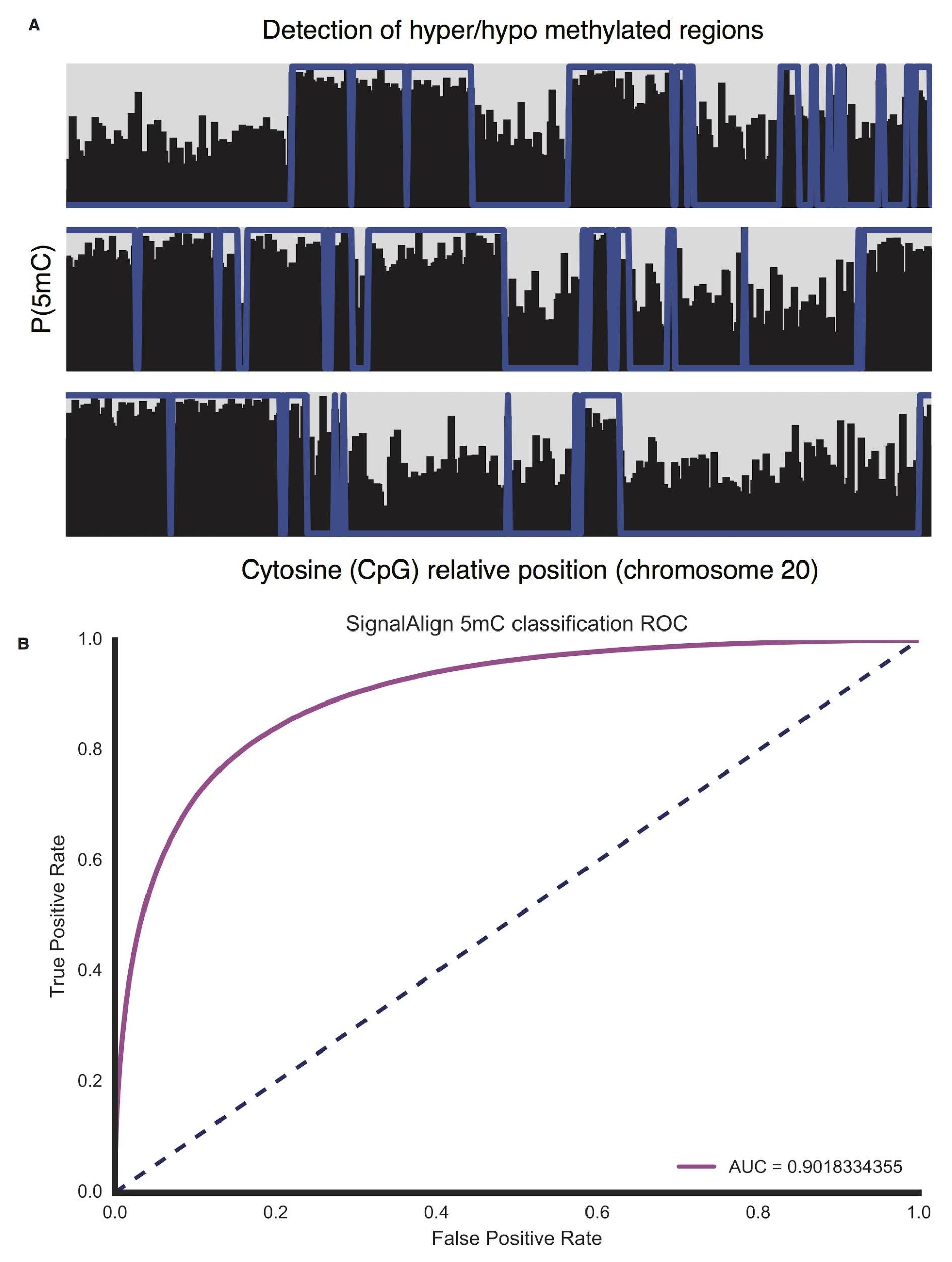

Supplement: Methylation — A) Native DNA methylation detection on a selected portion of chromosome 20. Individual plots show 500 called cytosine bases ordered along chromosome 20. Total marginal probability of methylation is shown as black bar. High-confidence methylation calls from ENCODE (ENCSR890UQO), blue line, were filtered for positions where all reads called methylated or not methylation to remove ambiguity. Cytosine calls were filtered to only sites with coverage >= 10 reads in both data sets. B) Receiver operating characteristic (ROC) plot describing SignalAlign as a binary classifier for individual 5-methyl cytosine detection (n=658,514).. [file 41587_2018_Article_BFnbt4060_Fig14_ESM.jpg]

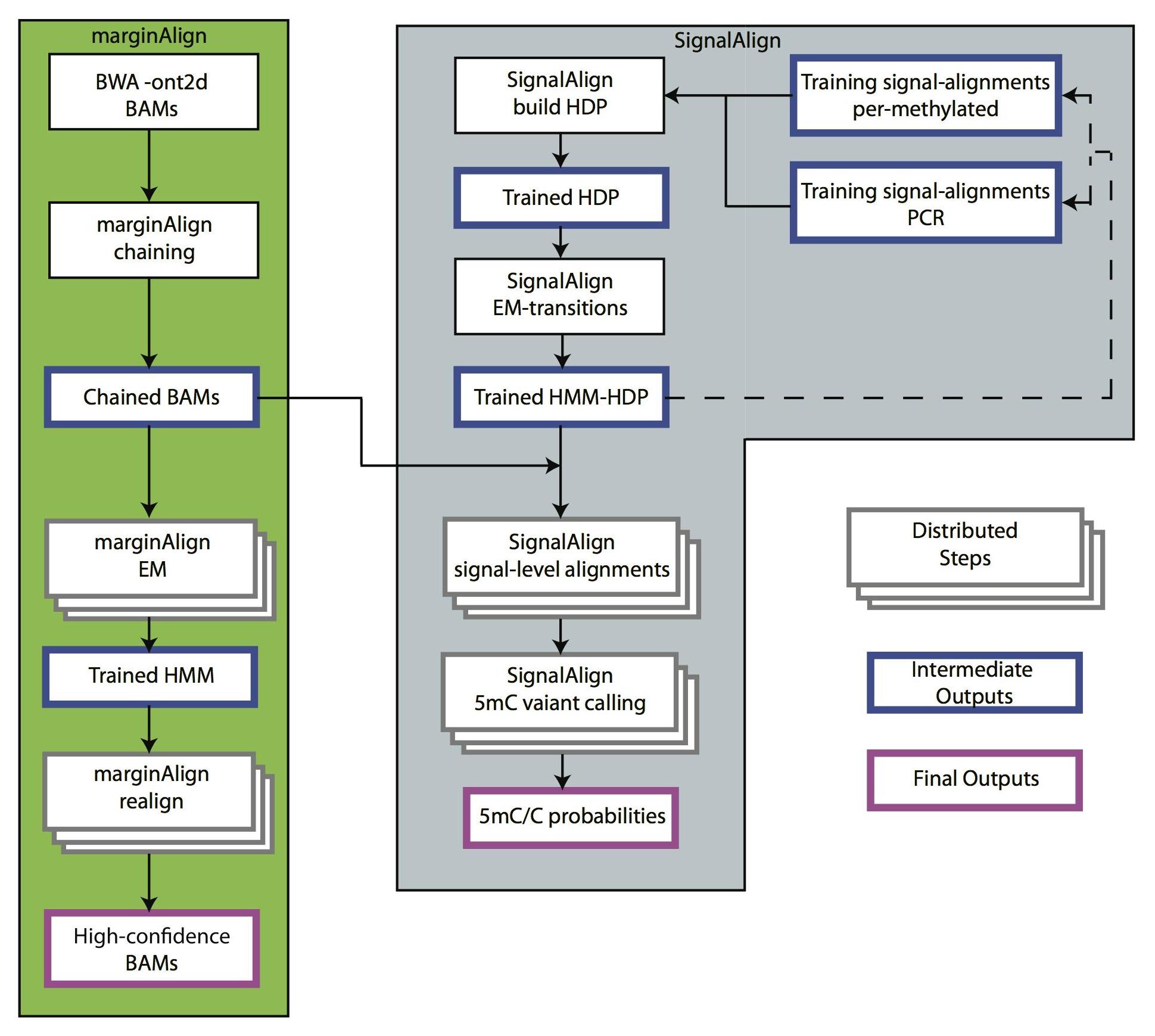

Supplement: marginAlign/SignalAlign Work Flow — Workflow chart describing marginAlign and SignalAlign. All distributed steps were implemented as part of a Toil-pipeline to be run in the cloud. Dotted lines represent repeating steps (iterations). The pipeline was repeated twice to validate the result. [file 41587_2018_Article_BFnbt4060_Fig15_ESM.jpg]

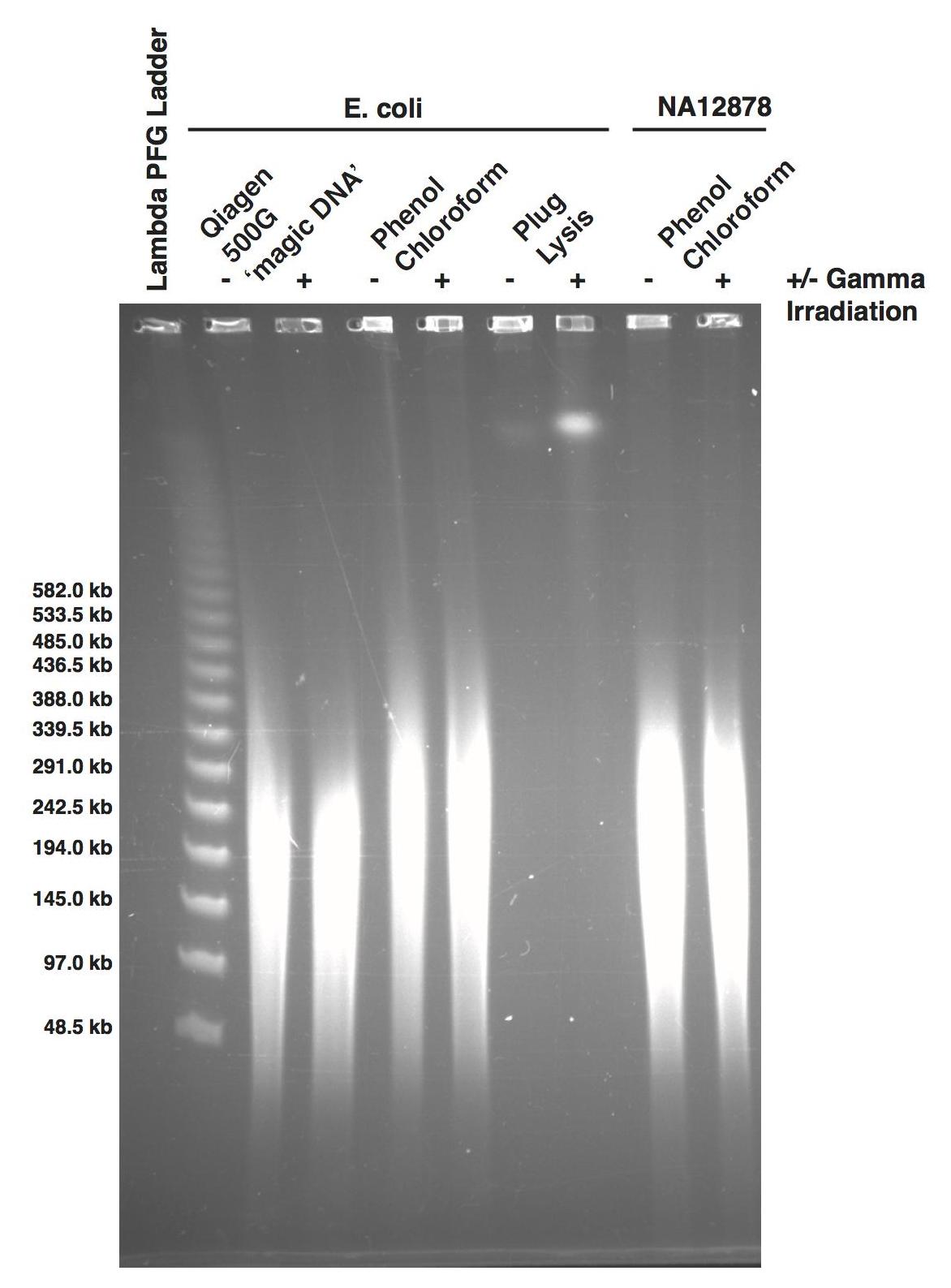

Supplement: Ultra-long reads DNA extraction — Pulsed-field gel showing fragment sizes of; E. coli MG1655 DNA extracted using the Qiagen Genomic DNA buffer set and a Qiagen 500G column following the protocol for bacteria (lanes 2 and 3), E. coli MG1655 DNA extracted using the Sambrook and Russell phenol/chloroform protocol described in the methods section (lanes 4 and 5), E. coli MG1655 DNA extracted using a plug lysis method to preserve intact chromosomes (lanes 6 and 7) and Human NA12878 DNA extracted using the Sambrook and Russell phenol/chloroform protocol described in the methods section (lane 8 and 9). For each pair of samples one was irradiated with approximately 35 Gray ionising radiation to introduce double-strand breaks, this improves the intensity of the band representing the 4.6 Mb E. coli MG1655 chromosome. A 1.2% PFG agarose gel made with 0.5% TBE and run on a Bio-Rad CHEF Mapper at 14°C for 20 hours 46 minutes with a two-state 120° included angle, 6 V/cm gradient, initial switch time 0.64s and final switch time 1m 13.22s. The gel was ethidium bromide stained and imaged on a Bio-Rad Gel Doc XR system. [file 41587_2018_Article_BFnbt4060_Fig16_ESM.jpg]
