# Supplementary material for: Nanopore sequencing and assembly of a human genome with ultra-long reads
Source: Nat Biotechnol. 2018 Jan 29;36(4):338–45. doi: 10.1038/nbt.4060 (PMC5889714; doi:10.1038/nbt.4060)
Supplement: Supplementary file 13 — Life Sciences Reporting Summary (PDF 161 kb) [file 41587_2018_BFnbt4060_MOESM33_ESM.pdf]

nature research

Corresponding author(s): Dr Matthew Loose, Prof Nicholas Loman

☐ Initial submission ☐ Revised version ☒ Final submission

Life Sciences Reporting Summary

Nature Research wishes to improve the reproducibility of the work that we publish. This form is intended for publication with all accepted life science papers and provides structure for consistency and transparency in reporting. Every life science submission will use this form; some list items might not apply to an individual manuscript, but all fields must be completed for clarity.

For further information on the points included in this form, see Reporting Life Sciences Research. For further information on Nature Research policies, including our data availability policy, see Authors & Referees and the Editorial Policy Checklist.

Experimental design

1. Sample size

Describe how sample size was determined.	A single sample was sequenced and so this is not applicable.

2. Data exclusions

Describe any data exclusions.	No data were excluded from the study.

3. Replication

Describe whether the experimental findings were reliably reproduced.	The experiments were carried out at five distinct sites. As a single sample was being sequenced with data combined together, replication was not required.

4. Randomization

Describe how samples/organisms/participants were allocated into experimental groups.	As a single sample was sequenced, no randomization was required in this study.

5. Blinding

Describe whether the investigators were blinded to group allocation during data collection and/or analysis.	No blinding was required for this study.

Note: all studies involving animals and/or human research participants must disclose whether blinding and randomization were used.

6. Statistical parameters

For all figures and tables that use statistical methods, confirm that the following items are present in relevant figure legends (or in the Methods section if additional space is needed).

n/a	Confirmed	
☐	☒	The exact sample size (n) for each experimental group/condition, given as a discrete number and unit of measurement (animals, litters, cultures, etc.)
☐	☒	A description of how samples were collected, noting whether measurements were taken from distinct samples or whether the same sample was measured repeatedly
☐	☒	A statement indicating how many times each experiment was replicated
☐	☒	The statistical test(s) used and whether they are one- or two-sided (note: only common tests should be described solely by name; more complex techniques should be described in the Methods section)
☐	☒	A description of any assumptions or corrections, such as an adjustment for multiple comparisons
☐	☒	The test results (e.g. P values) given as exact values whenever possible and with confidence intervals noted
☐	☒	A clear description of statistics including central tendency (e.g. median, mean) and variation (e.g. standard deviation, interquartile range)
☐	☒	Clearly defined error bars

See the web collection on statistics for biologists for further resources and guidance.

Software

Policy information about availability of computer code

7. Software

Describe the software used to analyze the data in this	All software used for data analysis are fully described in the materials and methods

June 2017

Nature Biotechnology: doi:10.1038/nbt.4060

study.

of the manuscript. All custom computer code is deposited in GitHub in appropriate repositories as described in the materials and methods. These are also linked from the main project GitHub page:
https://github.com/nanopore-wgs-consortium/NA12878

Software and versions used:

Oxford Nanopore Technologies:
MinKNOW 1.3.24 (ONT)
Metrichor (ONT)
Scrappie (https://github.com/nanoporetech/scrappie commit 2d5f7883a31152cf75ff77a060c751288f74e972) (ONT)
Albacore v 0.8.4 (ONT)

Nanopore Custom Tuning Scripts (Supplementary Code 1)
SVTyper (Supplementary Code 2 - http://github.com/tomsasani/svtyper commit d70de9c)
SignalAlign - https://github.com/ArtRand/toil-signalAlign
MarginAlign - https://github.com/ArtRand/toil-marginAlign
Poredb - https://github.com/nickloman/poredb
minControl - https://github.com/minoTour/minoTour
Comparative Annotation Toolkit (CAT) - https://github.com/ComparativeGenomicsToolkit/Comparative-Annotation-Toolkit commit c9503e7
Pilon 1.21 - https://github.com/skoren/PilonGrid
sdust - https://github.com/lh3/minimap commit 17d5bd12290e0e8a48a5df5afaeaef4d171aa133
Canu v1.4 - https://github.com/marbl/canu r8120 and r8006 (4a7090bd17c914f5c21bacbebf4add163e492d54)

For manuscripts utilizing custom algorithms or software that are central to the paper but not yet described in the published literature, software must be made available to editors and reviewers upon request. We strongly encourage code deposition in a community repository (e.g. GitHub). *Nature Methods* guidance for providing algorithms and software for publication provides further information on this topic.

▸ Materials and reagents

Policy information about availability of materials

8. Materials availability

Indicate whether there are restrictions on availability of unique materials or if these materials are only available for distribution by a for-profit company.

This study uses NA12878 cell line and DNA which is supplied by Coriell and is approved for genome sequencing governed by the Coriell Institutional Review Board ("Coriell IRB") in accordance with DHHS regulations (45 CFR Part 46).

9. Antibodies

Describe the antibodies used and how they were validated for use in the system under study (i.e. assay and species).

No antibodies were used.

10. Eukaryotic cell lines

a. State the source of each eukaryotic cell line used.

This study uses NA12878 cell line and DNA which is supplied by Coriell and is approved for genome sequencing governed by the Coriell Institutional Review Board ("Coriell IRB") in accordance with DHHS regulations (45 CFR Part 46) and is not considered human subjects research.

b. Describe the method of cell line authentication used.

Purchased from validated source and sequenced. Source validates cells as described here: https://www.coriell.org/0/pdf/CC_Process_Flow.pdf

c. Report whether the cell lines were tested for mycoplasma contamination.

Cells are routinely screened for mycoplasma and mycoplasma contamination would be detectable via sequencing.

d. If any of the cell lines used are listed in the database of commonly misidentified cell lines maintained by ICLAC, provide a scientific rationale for their use.

No commonly mis-identified cell lines were used.

Nature Biotechnology: doi:10.1038/nbt.4060

Animals and human research participants

Policy information about studies involving animals; when reporting animal research, follow the ARRIVE guidelines

11. Description of research animals

Provide details on animals and/or animal-derived materials used in the study.	No animals were used in this study.

Policy information about studies involving human research participants

12. Description of human research participants

Describe the covariate-relevant population characteristics of the human research participants.	This study uses NA12878 cell line and DNA which is supplied by Coriell and is approved for genome sequencing governed by the Coriell Institutional Review Board ("Coriell IRB") in accordance with DHHS regulations (45 CFR Part 46) and is not considered human subjects research.

Nature Biotechnology: doi:10.1038/nbt.4060
