# Supplementary material for: Nanopore sequencing and assembly of a human genome with ultra-long reads
Source: Nat Biotechnol. 2018 Jan 29;36(4):338–45. doi: 10.1038/nbt.4060 (PMC5889714; doi:10.1038/nbt.4060)
Supplement: Supplementary file 14 — Supplementary Tables 1–4, 6–9, and 11 (PDF 630 kb) [file 41587_2018_BFnbt4060_MOESM34_ESM.pdf]

Supplementary Tables

Supplementary Table 1 - Summary sequencing statistics

Summary statistics for every flowcell used in this study.

Flowcell ID	Number of Reads	Bases Sequenced	Date	Centre	Sample Type	Kit	Pore
FAB23716	356209	1409812422	14/07/16	UBC	DNA	Rapid	R9
FAB39088	658224	3287994454	19/09/16	Notts	DNA	Ligation	R9.4
FAB39075	466329	2439355478	20/09/16	UBC	DNA	Ligation	R9.4
FAB39043	436976	2273008592	23/09/16	Bham	DNA	Ligation	R9.4
FAB42706	430660	1966505502	12/10/16	UBC	DNA	Ligation	R9.4
FAB41174	117057	687394987	13/10/16	Bham	DNA	Ligation	R9.4
FAB42260	267644	1399557161	13/10/16	UBC	DNA	Ligation	R9.4
FAB42316	572838	3275026637	14/10/16	Notts	DNA	Ligation	R9.4
FAB42205	317654	1686630108	14/10/16	Notts	DNA	Ligation	R9.4
FAB42804	16669	75062609	14/10/16	Bham	DNA	Ligation	R9.4
FAB42561	233678	1520513556	19/10/16	Notts	DNA	Ligation	R9.4
FAB42473	644869	3357548938	19/10/16	UBC	DNA	Ligation	R9.4
FAB42395	38291	179704035	20/10/16	Norwich	DNA	Ligation	R9.4
FAB42476	435158	2363036522	27/10/16	UBC	DNA	Ligation	R9.4
FAB42451	817629	4530477841	28/10/16	Notts	DNA	Ligation	R9.4
FAB42704	276152	1750149482	28/10/16	UBC	DNA	Ligation	R9.4
FAB42828	33527	163405138	01/11/16	Norwich	DNA	Ligation	R9.4
FAB42810	322058	2020615256	02/11/16	Norwich	DNA	Ligation	R9.4
FAB42798	193551	1339441522	03/11/16	Norwich	DNA	Ligation	R9.4
FAB45280	128234	799554798	11/11/16	Norwich	DNA	Ligation	R9.4
FAB46664	491346	2038018797	15/11/16	UBC	DNA	Ligation	R9.4
FAB46683	72605	286275511	17/11/16	Bham	DNA	Ligation	R9.4
FAB45332	530938	2864140853	17/11/16	UBC	DNA	Ligation	R9.4
FAB44989	558224	3443824633	18/11/16	UCSC	DNA	Ligation	R9.4

FAB43577	426941	2539015084	18/11/16	UCSC	DNA	Ligation	R9.4
FAB45321	299174	2584017112	22/11/16	Notts	Cells	Ligation	R9.4
FAF01441	254705	2203636947	22/11/16	Bham	Cells	Ligation	R9.4
FAF01169	339447	2913892142	22/11/16	Bham	Cells	Ligation	R9.4
FAB45277	53547	445641679	22/11/16	Notts	Cells	Ligation	R9.4
FAF01127	632728	4972081712	25/11/16	Bham	Cells	Ligation	R9.4
FAF01132	689781	5455971336	25/11/16	Bham	Cells	Ligation	R9.4
FAB45271	472656	3689043164	28/11/16	Notts	Cells	Ligation	R9.4
FAB45321	123037	1043504055	28/11/16	Notts	Cells	Ligation	R9.4
FAB49914	309175	2841008085	28/11/16	Notts	Cells	Ligation	R9.4
FAB49712	632158	4906148911	28/11/16	Bham	Cells	Ligation	R9.4
FAF01253	471698	3695661984	28/11/16	Bham	Cells	Ligation	R9.4
FAB49164	746333	4438258089	06/12/16	UCSC	DNA	Ligation	R9.4
FAF04090	91304	1213584440	09/12/16	Bham	Cells	Rapid	R9.4
FAB49908	224380	3141600861	09/12/16	Bham	Cells	Rapid	R9.4
FAF15665	82138	1806857522	10/03/17	Notts	Cells	Ultrareads	R9.4
FAF13748	53723	1252868852	10/03/17	Notts	Cells	Ultrareads	R9.4
FAF10039	41385	848632752	01/03/17	Bham	Cells	Ultrareads	R9.4
FAF09968	19674	594496244	03/03/17	Bham	Cells	Ultrareads	R9.4
FAF09277	73755	1987434656	03/06/17	Bham	Cells	Ultrareads	R9.4
FAF14035	75692	1831031405	08/03/17	Notts	Cells	Ultrareads	R9.4
FAF15694	61227	1533616061	06/03/17	Bham	Cells	Ultrareads	R9.4
FAF09713	65142	1639658993	07/03/17	Bham	Cells	Ultrareads	R9.4
FAF18554	270189	2730589684	06/03/17	UBC	Cells	Rapid	R9.4
FAF15630	9663	322753214	09/03/17	Notts	Cells	Ultrareads	R9.4
FAF09640	72936	1496943560	07/03/17	Bham	Cells	Ultrareads	R9.4
FAF09701	68169	1731054841	03/03/17	Bham	Cells	Ultrareads	R9.4
FAF15586	71155	1750584936	08/03/17	Bham	Cells	Ultraread	R9.4
FAF05869	451020	3613667827	08/03/17	UBC	Cells	Ligation	R9.4

Supplementary Table 2 - Summary alignment statistics by Flowcell
Summary alignment statistics (bwa mem) for each flow cell, excluding ultra-long reads.

Flow Cell ID	Number of Sequences	Mapped Reads	Mapped MQ0	Unmapped	Bases Mapped	Avg Length
FAB23716	356209	319259	26702	36950	1165998694	3957
FAB39088	658224	613044	35394	45180	3007307322	4995
FAB39075	466329	425117	28167	41212	2146453407	5230
FAB39043	436976	415389	21043	21587	2113140439	5201
FAB42706	430660	375374	17378	55286	1867123361	4566
FAB41174	117057	114520	4186	2537	652217119	5872
FAB42260	267644	246982	15624	20662	1263089767	5229
FAB42804	16669	13311	1755	3358	53666089	4503
FAB42316	572838	512994	18985	59844	3100596254	5717
FAB42205	317654	282502	12561	35152	1601397762	5309
FAB42561	233678	225141	10255	8537	1420740185	6506
FAB42473	644869	611138	32539	33731	3112342902	5206
FAB42395	38291	36477	2059	1814	167168840	4693
FAB42476	435158	416969	20908	18189	2214880871	5430
FAB42451	817629	779328	36986	38301	4178966543	5540
FAB42704	276152	263722	12926	12430	1619875186	6337
FAB42828	33527	27843	2442	5684	146819837	4873
FAB42810	322058	305070	16802	16988	1808343119	6274
FAB42798	193551	185739	8749	7812	1232035338	6920
FAB45280	128234	122219	6336	6015	743280816	6235
FAB46664	491346	456247	27622	35099	1862427349	4147
FAB46683	72605	64739	5307	7866	269213160	3942
FAB45332	530938	497862	26392	33076	2620752139	5394
FAB43577	426941	410137	19835	16804	2344990054	5946
FAB44989	558224	536572	25936	21652	3161900821	6169
FAF01169	339447	315489	16481	23958	2677881316	8584
FAF01441	254705	238834	12458	15871	2010117898	8651

FAB45277	53547	51957	2132	1590	426639054	8322
FAB45321	299174	283355	15165	15819	2366003310	8637
FAF01127	632728	605633	27192	27095	4640355789	7858
FAF01132	689781	655357	33564	34424	4966810089	7909
FAB49712	632158	612752	26264	19406	4594356245	7760
FAF01253	471698	454434	20639	17264	3430678969	7834
FAB45321	123037	118311	5891	4726	952851126	8481
FAB49914	309175	296250	12281	12925	2673848960	9188
FAB45271	472656	450702	20148	21954	3468377327	7804
FAB49164	746333	718351	32664	27982	4107087899	5946
FAB49908	224380	211060	11903	13320	2898563539	14001
FAF04090	91304	83164	6072	8140	1085757398	13291

Supplementary Table 3 - Summary alignment statistics by chromosome

(Excluding ultra-long reads)

Chromosome	Number of Mapped Reads	Number of Mapped Reads (MQ0)	Number of Bases Mapped	Avg Length
Chr 1	1075867	43397	6829526262	6744
Chr 2	1062314	31802	6755642896	6842
Chr 3	858643	24189	5487703898	6757
Chr 4	845677	30723	5395140705	6890
Chr 5	774613	23499	4953273570	6821
Chr 6	723047	24496	4618883250	6762
Chr 7	696473	28231	4382999832	6772
Chr 8	617988	23361	3968911801	6844
Chr 9	539660	25898	3428430670	6764
Chr 10	594688	20787	3805443564	6845
Chr 11	583055	17748	3710684724	6855

Chr 12	586663	17891	3734922623	6840
Chr 13	440615	17662	2844212242	6904
Chr 14	383777	15752	2439119767	6713
Chr 15	359853	19556	2268233023	6838
Chr 16	386401	22680	2425913744	6787
Chr 17	369036	22907	2302471086	6661
Chr 18	339094	13053	2172098564	6807
Chr 19	257039	10926	1472760724	6266
Chr 20	291960	13226	1829244829	6659
Chr 21	192383	24988	1207807437	6792
Chr 22	172934	10514	1041347396	6665
Chr X	658347	28769	4210769167	7076
Chr Y	23378	5292	133803203	7869
Chr M	59363	658	91949786	1628

Supplementary Table 4 - Read Length Metrics by DNA Input

Summary read length metrics subdivided by DNA preparation and sequencing library preparation method.

Input DNA	Number of Reads	Median Read Length	Mean Read Length	Total Bases	Read N50
Ligation Library, Cell DNA	4278106	7140	8123	34750607127	12112
Ligation Library, Cell DNA (Albacore/ MinKNOW 1.4 Control)	451020	4463	8012	3613667827	13920
Ligation Library, Coriell DNA	9233585	3853	5493	50724515583	9136
Rapid Kit, Cell DNA	315684	6800	13796	4355185301	30397
Rapid Kit, Cell DNA (Albacore/ MinKNOW 1.4 Control)	270189	4073	10106	2730589684	24848

Rapid Kit, Coriell DNA	356209	2375	3958	1409812422	6978
Ultralong reads Protocol, Cell DNA	694659	3488	24179	16795933036	99790

Supplementary Table 5 Kmer Count Summaries.

Summary containing kmer counts with respect to chromosome 20 for each of the base callers used in this study: See Supplementary_Table5_kmer.xlsx

Supplementary Table 6 Structural Variants

Summary of structural variants observed in the Nanopore only and Nanopore polished canu assemblies.

		PacBio		Nanopore		Nanopore Polished	
**SV Type**	**Size Range (bp)**	**Count**	**Total bp**	**Count**	**Total bp**	**Count**	**Total bp**
Insertion	50-500	2042	306272	3365	610141	2446	383069
	500-10000	319	618769	535	767711	394	681803
	**Total:**	2361	925041	3900	1377852	2840	1064872
Deletion	50-500	1645	290106	26853	4718511	18359	3358506
	500-10000	1079	3142086	1938	2047493	2438	2528925
	**Total:**	2724	3432192	28791	6766004	20797	5887431
Tandem expansion	50-500	1431	316231	1199	358827	1782	422450
	500-10000	778	1024584	1067	1216138	1068	1285297
	**Total:**	2209	1340815	2266	1574965	2850	1707747
Tandem contraction	50-500	685	114672	629	96955	1286	209359
	500-10000	159	251549	75	158553	186	345439
	**Total:**	844	366221	704	255508	1472	554798
Repeat expansion	50-500	241	57575	1961	389003	1289	251816
	500-10000	373	894444	469	688134	420	707130
	**Total:**	614	952019	2430	1077137	1709	958946

Repeat contraction	50-500	368	104240	24178	5467319	14276	3258222
	500-10000	1627	3718349	6882	6930904	3129	2810950
	**Total:**	1995	3822589	31060	12398223	17405	6069172
**Totals for all variants:**		10747	10838877	69151	23449689	47073	16242966

Supplementary Table 7 - SRA accessions for comparative annotation. List of SRA accessions for RNA-seq data used to guide comparative annotation process.

**Project Accession**	**SRA Accessions**	**Tissues**
PRJEB6971	ERR579132	Ovary
	ERR579133, ERR579135, ERR579139, ERR579145	Tonsil
	ERR579134, ERR579136, ERR579150, ERR579155	Fallopian Tube
	ERR579137	Placenta
	ERR579138	Endometrium
	ERR579140, ERR579147, ERR579151	Rectum
	ERR579141, ERR579142, ERR579143, ERR579149, ERR579151	Skeletal Muscle
	ERR579144	Liver
	ERR579146	Fat
	ERR579148	Colon
	ERR579154	Lung
	ERR579153	Smooth Muscle

Supplementary Table 8 - GraphMap Alignment Statistics

Summary alignment statistics (GraphMap) for each flow cell, including ultra-long reads.

Flow Cell ID	Number of Sequences	Mapped Reads	Unmapped	Bases Mapped	Avg Length
FAF10039	47446	23128	24318	667656276	28868
FAF09701	68169	32100	36069	1479270745	46083
FAF15665	84288	34226	50062	1597993761	46689
FAF09968	17524	10188	7336	443108888	43493
FAF09277	73755	41754	32001	1709299350	40937
FAF15694	61227	33146	28081	1341056424	40459
FAF09640	72936	37069	35867	1285502606	34679
FAF09713	65142	38879	26263	1407531224	36203
FAF15586	68186	32820	35366	1491306664	45439
FAF15630	9663	7086	2577	282238538	39830
FAF13748	53723	31047	22676	1099141612	35403
FAF14035	75692	39804	35888	1503376661	37769
FAB45271	472656	406691	65965	3314072035	8149
FAB45321	299174	245380	53794	2249171828	9166
FAB42316	572838	477558	95280	3058327902	6404
FAB45280	128234	109110	19124	696764382	6386
FAB44989	558224	482024	76200	3011499106	6248
FAB45321	123037	103270	19767	911283560	8824
FAB46664	491346	412952	78394	1787039440	4327
FAB39088	658224	553407	104817	2839325410	5131
FAB42828	33527	22135	11392	129043084	5830
FAB42798	193551	167706	25845	1180440415	7039
FAB42810	322058	268807	53251	1696713127	6312
FAF01127	632728	547723	85005	4425604598	8080
FAB42561	233678	206308	27370	1377358663	6676
FAB42205	317654	261747	55907	1568315755	5992

FAB43577	426941	370142	56799	2261278047	6109
FAB39043	436976	382402	54574	2026829908	5300
FAB49914	309175	266347	42828	2541235367	9541
FAF04090	91304	65645	25659	967221027	14734
FAB42476	435158	382131	53027	2140806786	5602
FAF01441	254705	208108	46597	1900156923	9131
FAB41174	117057	107747	9310	643269826	5970
FAB49164	746333	655827	90506	3949798956	6023
FAB42706	430660	350227	80433	1829831204	5225
FAB42260	267644	216192	51452	1221035191	5648
FAB42395	38291	33280	5011	160506136	4823
FAB42473	644869	554957	89912	2990969347	5390
FAB42451	817629	715365	102264	4022701305	5623
FAB23716	356209	263472	92737	1071097685	4065
FAF01169	339447	275736	63711	2539526236	9210
FAB39075	466329	371194	95135	1989101091	5359
FAB46683	72605	55639	16966	251376458	4518
FAB49908	224380	177511	46869	2675201854	15071
FAB45332	530938	451596	79342	2498402122	5532
FAB49712	632158	557647	74511	4396917975	7885
FAF01253	471698	409846	61852	3287940955	8022
FAB42804	16669	8324	8345	45639694	5483
FAF01132	689781	576530	113251	4682167262	8121
FAB45277	53547	47344	6203	408114396	8620
FAB42704	276152	238696	37456	1541840559	6459
FAF05869	451020	391538	59482	3240265604	8276
FAF18554	270189	226421	43768	2466492604	10893

Supplementary Table 9 - **Summary of HLA Typing**

Genotyping was performed based on MUMmer alignments of the HLA genes and CDS nucleotides fasta files downloaded from IPD-IMGT (Methods). Gene boundaries were identified according to the gene order as in HLA haplotyping nomenclature. A haplotype was identified as the minimal edit distance to a known sequence on both a gene and exon level. For DRB1 on haplotype A, we chose the gene level haplotype with highest gene coverage and lowest edit distance. Distance measured using exon 2 and 3 for MHC class I genes and exon 2 for MHC class II genes. The G group was called by the definitions at http://hla.alleles.org/alleles/g_groups.html. If all matches agreed on a G group, it was reported as the called type for the assembly.

Haplotype	Haplotig	Locus	True HLA type (phased)	Edit distance assembly vs. truth	Edit distance called allele vs. truth
A					
	tig00000001_A_pilon	HLA-A	01:01:01G	9	1
	tig00000008_A_pilon	HLA-B	08:01:01G	1	0
	tig00000008_A_pilon	HLA-C	07:01:01G	1	1
	tig00000022_A_pilon	HLA-DQA1	05:01:01G	0	0
	tig00000049_A_pilon	HLA-DQB1	02:01:01G	0	0
	tig00000049_A_pilon	HLA-DRB1	03:01:01G	na	na
B					
	tig00000003_B_pilon	HLA-A	11:01:01G	0	0
	tig00000010_B_pilon	HLA-B	56:01:01G	4	0

	tig00000012_B_pilon	HLA-C	01:02:01G	1	0
	tig00000012_B_pilon	HLA-DQA1	01:01:01G	0	0
	tig00000031_B_pilon	HLA-DQB1	05:01:01G	0	0
	tig00000031_B_pilon	HLA-DRB1	01:01:01G	11	1

Supplementary Table 10 - Summary of gap closures within the canu reference assembly and ultra-long reads from this study which span them: See Supplementary_Table10_gaps.xlsx.

Chromosome GRCh38 (GCA_000001305.2) coordinates, chromosome banding information, and flanking assembled contigs are listed for each gapped region. A region is considered "closed" if it is spanned by our canu assembly and if the sequence structure observed at the assembly closure is supported by at least one 100 kb + read. The assembled contig ID from this study observed to close the gap is listed as Canu Assembly. The total number of bases inserted from the gapped region are represented as Gap size (bp). New, inserted sequences are primarily repetitive (simple sequence repeats and transposable elements), as determined by RepeatMasker. Long reads that span the gaps and provide concordant evidence for the inserted sequence in the assembly are provided. Alignments of the long reads to the assembled contigs was performed using minimap2 [37] .

Supplementary Table 11 - Telomere Chromosome Assignments

Characterization of telomeric repeat lengths using ultra long reads. Ultra long read alignments were determined using minimap2-2.11 (r341; github.com/lh3/minimap2) with default parameters. Telomere repeat structure on each read was determined using HMM profile modeling of the published telomere tract of repeats M19947.1, with the total number of estimated bases listed as “Est TEL Repeat”.

Tel	Unique Read Id	CHR Start	CHR End	RD LEN	Est TEL Repeat
4p	4a6fa80f-3ba3-4a23-92ac-02bfb2041c7d	10248	261509	240185	9964
5p	aa9292b3-abaa-4fe3-af27-e2a43d055cfd	12465	173761	159637	6451
5q	edde5135-5e84-4389-967f-e1ab09a86b52	181367404	181476277	109110	2791
9p	ffd32c96-36aa-441e-9cc2-ab233d65e15e	11264	374849	336855	1712
10p	62e38601-5a61-430e-b047-84964d1757d2	10778	172328	161402	10520
10q	05e99c44-b96e-4ebe-a83c-f3eed6c27a39	133591791	133786895	158136	7227
12q	e5be0ad4-173f-4a71-96df-ef7868d40938	133104863	133259321	151656	6268
16p	29e3cce4-c451-496c-ad8c-acbca82c0947	10581	142370	129255	7297
16q	35ac4d8b-956e-48e2-92a4-9232a9b44602	90066445	90228276	162410	7037
18p	61f96a3b-43b8-4218-890a-eaff228aa4c9	10929	405285	370861	1573

19p	d42cbbaa-ad9e-4401-b6cc-db6eeebdb876	60007	209331	151977	8457
19q	ee33aec6-cbd2-4e3a-8401-933382c28f88	58255408	58600576	327135	5436
19q	c05710ce-b225-410d-86b1-877a130e2dc6	58405058	58605642	187407	6919
19q	5d1474fd-e8de-49ff-8199-4f5a575e6fb8	58475094	58605641	130915	8167
21q	cc14a9c9-0d5c-4198-ac9d-04ffa4968798	46506906	46698746	180969	9108
21q	12fc1c4d-d038-482f-bdb1-cfd172045692	46555114	46699882	138700	4939
